# Multifaceted Function of MicroRNA-299-3p Fosters an Antitumor Environment Through Modulation of Androgen Receptor and VEGFA Signaling Pathways in Prostate Cancer

**DOI:** 10.1038/s41598-020-62038-3

**Published:** 2020-03-20

**Authors:** Kavya Ganapathy, Stephen Staklinski, Md Faqrul Hasan, Richard Ottman, Thomas Andl, Anders E. Berglund, Jong Y. Park, Ratna Chakrabarti

**Affiliations:** 1 Burnett School of Biomedical Sciences, University of Central Florida, Orlando, FL USA; 2 Department of Biostatistics and Bioinformatics, Moffitt Cancer Center, Tampa, FL USA; 3 Department of Cancer Epidemiology, Moffitt Cancer Center, Tampa, FL USA

**Keywords:** Prostate cancer, miRNAs

## Abstract

Prostate cancer (PCa) is one of the most common cancers to affect men worldwide. Androgen receptor (AR) signaling is central to PCa and PCa therapy. MicroRNAs (miRNAs) play crucial roles in the regulation of prostate cancer through modulation of signaling pathways. In the present study, we illustrate the functional significance and therapeutic benefit of miR-299-3p, an AR targeting microRNA, in PCa progression. We noted loss of expression of miR-299-3p in prostate tumors compared to noncancerous prostate tissues. Replenishment of miR-299-3p in C4-2B, 22Rv-1 and PC-3 cells contributed to cell cycle arrest, reduced proliferation, migration and increased expression of apoptotic markers. Additionally, overexpression of miR-299-3p induced a reduction of AR, PSA and VEGFA expression. AGO-RNA pulldown experiment showed enrichment of AR, VEGFA and miR-299-3p in C4-2B cells overexpressing miR-299-3p. miR-299-3p overexpression also inhibited epithelial mesenchymal transition, expression of Slug, TGF-β3, phospho-AKT and phospho-PRAS40, but increased expression of E-cadherin. Furthermore, miR-299 overexpression resulted in reduced tumor growth in xenograft models and increased drug sensitivity. Overall, this study has identified novel mechanisms of antitumor and antimigration function of miR-299-3p through modulation of AR and VEGFA signaling pathways which lead to improved drug sensitivity of PCa.

## Introduction

MicroRNAs (miRNAs) are a group of small regulatory RNAs that play critical roles in the regulation of gene expression^1^. MiRNAs can have a huge impact on a broad spectrum of biological processes such as cell differentiation, proliferation, apoptosis and carcinogenesis^2^. Abnormal miRNA expression has been observed in several cancer types and contributes to the phenotypic changes that are commonly associated with cancer hallmarks. Previous studies have shown an association between aberrant miRNA expression, cancer aggressiveness and development of drug resistance^3^. Furthermore, miRNA expression signatures may become valuable diagnostic and prognostic markers. As miRNAs target and regulate multiple genes, they can also be used as potential therapeutic tools to restore the balance between oncogenes and tumor suppressors in cancer cells^4^.

Currently, first-line treatment options for PCa include radical prostatectomy, often used in combination with radiation therapy and chemotherapy. For advanced PCa, blocking androgen receptor signaling by androgen deprivation therapy (ADT) is the dominant targeted therapy of choice^5^. Although most of the patients show an initial positive response, eventually their tumors develop resistance to ADT and progress to the more aggressive castration resistant prostate cancer (CRPC) stage^6^. Androgen independence develops due to different mechanisms adapted by the PCa cells to thrive in low androgen levels. These changes allow the CRPC cells to survive and evade apoptosis during treatment, thereby, causing the recurrence of the cancer^7^. MiRNAs often target a spectrum of genes and therefore could offer advantages over monotherapies targeting a single pathway. To exploit this characteristic of miRNAs, a better understanding of their involvement in PCa is necessary. A number of miRNAs are involved in progression from localized disease to aggressive PCa including miR-218^8^, -143^9^, -145^10^ and let7c^11^, whereas, restored expression of the miR-17-92a cluster miRNAs has tumor suppressor effects in metastatic PCa cells^12^.

MicroRNA-299 is located at the chromosome location 14q32.31 within a large cluster of miRNAs which has previously been implicated in prostate cancer. The data also suggested that the cluster of miRNAs at this specific chromosome location were involved in the progression of PCa to a more aggressive state^13^. Recently, published reports have shown that miR-299-3p exhibits a tumor suppressor role in other cancers by targeting multiple genes^14,15^. Similarly, some targets of miR-299-3p were reported such as *ABCE1* in lung cancer^16^, *OCT4* in breast cancer and fibrosarcoma^17^, *SHOC2* in thyroid cancer^18^, and AR in prostate cancer^19^ suggesting that restoring miR-299-3p expression in prostate cancer may have pleiotropic effects mediated by several target genes. However, a detailed functional characterization of miR-299-3p and the underlying mechanism in PCa progression through different targets is still missing.

In this study, we have explored the role of miR-299-3p in PCa by studying its effect on two different target genes, AR and VEGFA in AR-positive and -negative cell culture systems. We also studied the overall effect of miR-299-3p on different phenotypic characteristics associated with cancer progression including activation of signaling cascades, tumor growth and drug sensitivity using cell culture and xenograft models. Our data suggest that miR-299-3p is frequently downregulated in PCa cells and tissues and exerts a tumor suppressor role through the bimodal targeting of AR and VEGFA to inhibit different signaling cascades that are constitutively active in PCa.

## Results

### miR-299-3p shows reduced expression in prostate tumor tissues and cells

**Figure 1:**
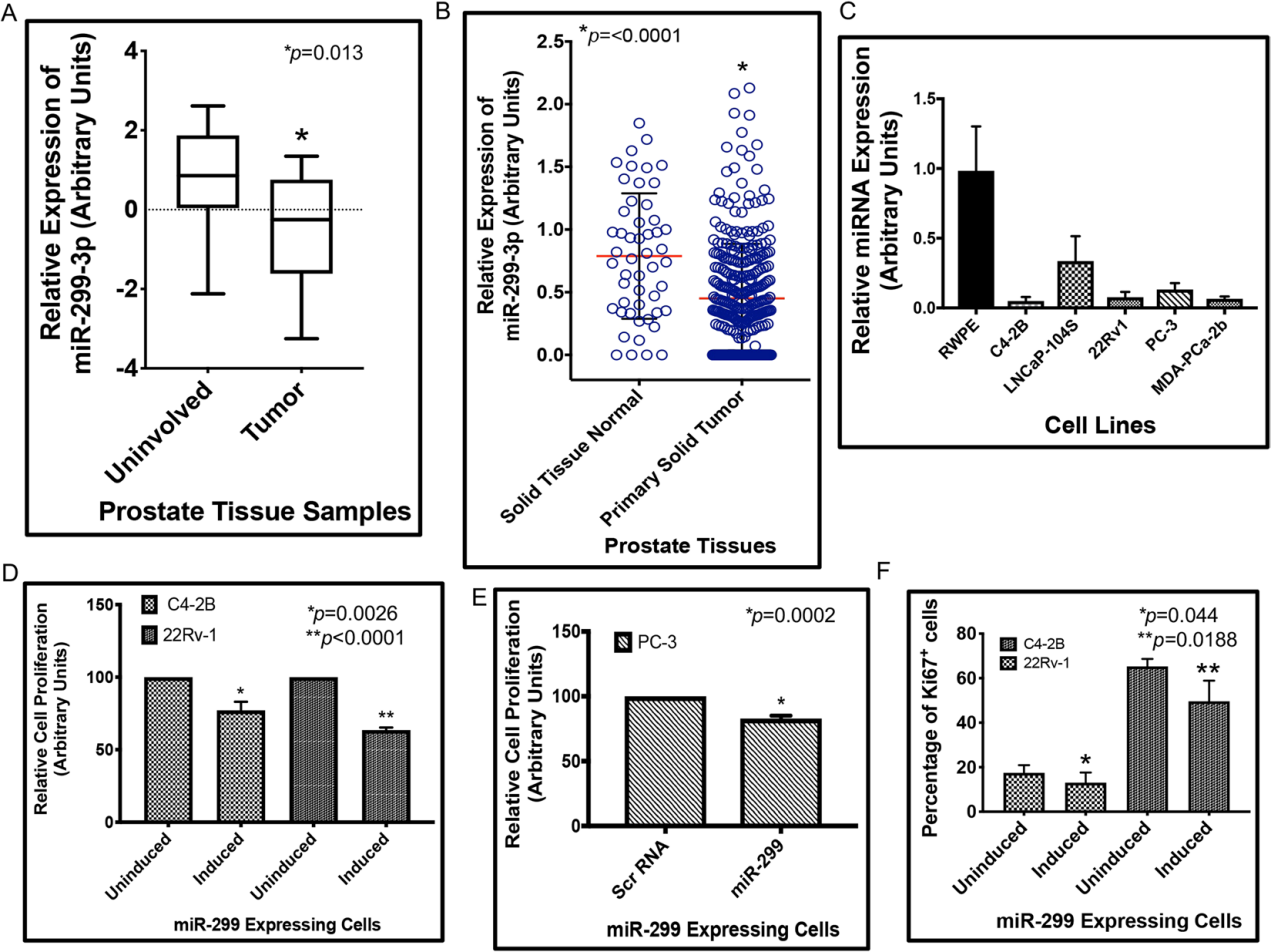
Endogenous miR-299-3p expression in PCa cell lines and tissues and miR-299-3p overexpression decreased cell proliferation. (**A**) Average fold change in expression of miR-299-3p prostate tumor tissues (n = 15) compared to matched uninvolved areas (15), and 3 additional tumor tissues. (**B**) TCGA database analysis showing significant loss of expression of miR-299-3p in prostate tumors compared to normal tissues. (**C**) Quantitative RT-PCR showing relative fold change in miR-299-3p expression in PCa cell lines compared to non-tumorigenic RWPE-1 cells. Raw data have been normalized to the mean of RNU43, U6 and U1 snRNA. (**D,E**) Cell proliferation assays showing significantly reduced cell growth in miR-299-3p overexpressing cells. Data represent mean ± standard deviation (SD) of at least three independent assays in triplicates. C4-2B and 22Rv-1 cells stably transfected (**D**) and PC-3 cells transiently transfected (**E)** with inducible DNA constructs for miR-299-3p precursor miRNA or scrambled (Scr) RNA (PC-3) were induced (PC-3 at 24 h post transfection) and cell proliferation at 48 hr were detected by MTS assays. (**F**) Analysis of Ki67^+^ cells upon immunofluorescence staining for Ki67 performed at 72 hr post induction showing significant reduction in Ki67^+^ C4-2B and 22Rv-1 cells overexpressing miR-299-3p.

To define the association of miR-299-3p, which is one of the few miRNAs that target AR, with progression of PCa, we first analyzed the expression pattern of miR-299-3p in macro-dissected PCa tissues. Selected patients were between 43-71 years of age and had undergone radical prostatectomy without any other prior treatments. Patients showed a presurgical PSA range of 4.3–87.4 and Gleason Score between 6–9. Patient criteria with clinical stages is presented in Table 1 in Supplementary data. Normalized fold change expression analysis showed down regulation (1.9-fold mean expression) of miR-299-3p in the tumor tissues compared to uninvolved areas (Fig. 1A). We did not observe any significant correlation with Gleason Scores. Further supporting our observation of reduced miR-299-3p expression, data from the The Cancer Genome Atlas Prostate Adenocarcinoma (TCGA PRAD) cohort showed a significantly lower expression of miR-299-3p in tumor tissue compared to normal tissue (Fig. 1B). Analysis of endogenous expression of miR-299-3p in non-tumorigenic (RWPE-1) and tumorigenic PCa cells showed reduced expression in all advanced and metastatic PCa cells compared to RWPE-1 cells (Fig. 1C). These observations prompted us to explore the functional significance of the reduced expression of miR-299-3p in PCa progression to an aggressive disease.

### Restoration of miR-299-3p expression reduces cell proliferation, cell cycle arrest and expression of cyclins

**Figure 2:**
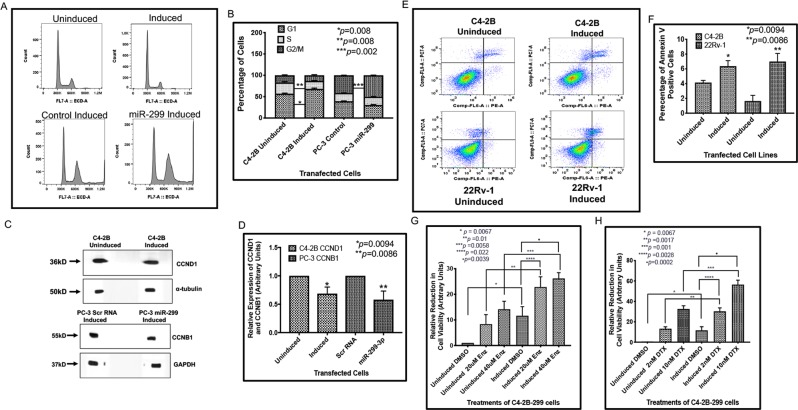
miR-299-3p induced cell cycle arrest and apoptosis, reduced expression of cyclins and improved drug sensitivity: All data show the mean ± SD of at least 3 independent experiments. (**A,B**) Two parameter histograms of cell cycle of induced C4-2B and PC-3 cells overexpressing miR-299-3p compared to uninduced or induced Scr RNA expressing cells. (**A**) Cell cycle analysis performed after 72 h of induction showing a G1 arrest (C4-2B, upper panel) and after 48 h of induction showing a G2/M arrest (PC-3, lower panel). (**B**) Comparative analysis of percentage of cells showing a significant increase in G1 phase cells and a significant decrease in S-phase cells, and a significant increase in G2/M phase cells compared to uninduced cells or induced Scr RNA expressing cells. (**C**) Representative images of Western Blot analysis of CCND1 (G1 marker) and CCNB1 (G2/M marker) and GAPDH or α-tubulin as the internal controls. (**D**) Comparative analysis of CCND1 and CCNB1 expression showing significant reduction in expression in induced C4-2B and PC-3 cells expressing miR-299-3p at 72 hr post induction, respectively, values were normalized to the internal controls. (**E**) Flow cytometric analysis of Annexin V expression in the induced C4-2B and 22Rv-1 cells overexpressing miR-299-3p compared to uninduced controls at 24 h after induction. (**F**) Quantification of Annexin V positive cells by FlowJo software showing increased percentage of apoptotic cells in the induced C4-2B-299 and 22Rv-1-299 cells compared to the uninduced control cells. (**G,H**) C4-2B-299 cells were induced or left uninduced and treated with ENZ and DTX and sensitivity to drugs was measured by MTS assays. DMSO was used as the vehicle control. Data represent mean ± SD of at least 3 independent experiments in triplicates. (**G**) Percent change in cell death in induced C4-2B-299 cells treated with 20 μM and 40 μM ENZ compared to uninduced cells and DMSO. (**H**) Percent change in cell death in induced C4-2B-299 cells treated with 2 nM and 10 nM DTX compared to uninduced cells and DMSO.

We generated the inducible cell lines C4-2B-299 and 22Rv-1-299 that overexpress miR-299-3p mature miRNA upon doxycycline treatment. We also used transiently transfected PC-3 cells that overexpress miR-299-3p (PC-3-299) upon induction compared to the control cells expressing scrambled (Scr) RNAs (Supplementary data Fig. S1A,B). We observed approximately a 10-fold increase of miR-299-3p in C4-2B and 22Rv-1 but PC-3 cells showed a much higher expression upon induction compared to control cells (Supplementary data Fig. S1C,D). Overexpression of miR-299-3p reduced cell proliferation in all three cell lines ranging from 17% to 37% compared to uninduced or control cells (Fig. 1D,E). Reduced cell proliferation was further confirmed by ~24% reduction in Ki67 expression in C4-2B-299 and 22Rv-1-299 cells induced with doxycycline compared to uninduced cells (Fig. 1F). Analysis of cell cycle progression showed a distinct G1 enrichment (20%) and S phase depletion (28%) in C4-2B-299 (Fig. 2A,B) induced cells compared to uninduced cells. A similar G1 arrest was noted for 22Rv-1-299 (8% increase in G1 and 16% depletion in S phase cells) (Supplementary data Fig. S2A,B). Induced PC-3-299 cells showed a significant increase in the percentage of cells in G2/M phase (22%) compared to induced control cells (Fig. 2A,B). The effect of miR-299-3p expression on cell cycle arrest was further supported by reduced expression of G1 cell cycle marker CCND1 in induced C4-2B-299 (Fig. 2C,D) (32%) and 22Rv-1-299 (Supplementary data Fig. S2C,D) (63%) cells compared to uninduced cells and G2/M phase marker CCNB1 (42% reduction) in induced PC-3-299 cells compared to induced control cells (Fig. 2C,D). However, computer algorithm based target analysis did not show any binding sites of miR-299-3p or 299-5p at the 3′UTR of CCND1 or CCNB1, which suggests an indirect mechanism of inhibition of CCND1 and CCNB1 expression in these cells.

### Expression of miR-299-3p induces apoptosis and improves drug sensitivity of AR-positive and AR-negative prostate cancer cells

It is possible that the effect of miR-299-3p expression on reduced cell growth could be associated with increased apoptosis, which led us to examine the onset of apoptosis in cells expressing miR-299-3p. We studied early apoptotic events by staining with Annexin V and noted that Annexin V positive cells were increased upon induction of C4-2B-299-3p (1.5-fold) and 22Rv-1-299-3p (4.2-fold) expression compared to uninduced cells (Fig. 2E,F). This finding prompted us to study the effect of miR-299-3p restoration on sensitivity to Enzalutamide (ENZ) as the parental C4-2B and 22Rv-1 cells are castration resistant^20,21^ and show low sensitivity to ENZ. Specifically, 22RV-1 cells express the AR-V7 splice variant^22^ in addition to full length AR, which is sufficient for the 22Rv-1 to show low sensitivity to ENZ that targets full-length AR^23^. In this study, we used C4-2B-299 and 22Rv-1-299 cells for treatment with ENZ (20 μM and 40 μM) with or without miR-299-3p induction with doxycycline. We noted reduced cell viability (2.7-1.8-fold) (8–23% for 20 μM, and 14–26% for 40 μM) in induced C4-2B-299 cells compared to uninduced cells upon ENZ treatments (Fig. 2G), showing an additive effect. There was also 1.9–2.2-fold reduction in cell viability in induced cells with and without ENZ treatments (Fig. 2G). For 22Rv-1 cells, the average reduction in cell viability was not pronounced upon ENZ treatment in uninduced cells (3.2% and 7.4% for 20 μM and 40 μM respectively) but a higher reduction in viability (3.8–1.9-fold) (3.2–12% for 20 μM and 7.4–14% for 40 μM) was noted in induced cells compared to uninduced cells upon ENZ treatments (Supplementary data Fig. S3A). No significant difference in cell viability could be noted in induced 22Rv-1-299 cells with or without ENZ treatment (Supplementary Fig. S3A), which suggests that ENZ treatment has no contribution in the decreased cell viability noted in induced cells.

Treatment with Docetaxel (DTX), on the other hand, showed significantly improved sensitivity of both C4-2B-299 and 22Rv-1-299 cells upon induction of miR-299-3p expression. A 13–30% and 33–56% reduction in cell viability showing a synergistic effect could be noted upon treatment with 2 nM and 10 nM DTX respectively of induced C4-2B-299 cells compared to uninduced cells (Fig. 2H). DTX treatment also reduced cell viability (2.5-fold for 2 nM and 4.7-fold for 10nM) compared to DMSO treatments in induced C4-2B-299 cells (Fig. 2H). A significant reduction in cell viability 15–33% (2 nM DTX) and 31–51% (10 nM DTX) showing a synergistic effect were noted in induced 22Rv-1-299 cells also upon DTX treatment compared to uninduced cells (Supplementary Fig. S3B). A 2.8-4.8-fold decrease in cell viability resulted from DTX treatment compared to DMSO in induced cells (Supplementary data Fig. S3B).

Similarly, treatment with DTX in AR-negtive PC-3 cells showed increased sensitivity of cells upon expression of miR-299-3p. Treatment with 10 nM and 25 nM DTX reduced cell viability by 31% and 55%, respectively in induced miR-299-3p PC-3 cells compared to induced control (Supplementary data Fig. S3C). Furthermore, cell viability was reduced by 15-40% in miR-299-3p expressing PC-3 cells with and without DTX (Supplementary data Fig. S3C).

To rule out the possibility that the effect of ENZ may be cell-line specific, we tested the combinatorial effect of miR-299-3p expression and ENZ treatment on cell viability in AR-sensitive LNCaP-104S cells. Similar to C4-2B cells, we noted reduced cell viability in induced miR-299-3p LNCaP-104S cells compared to induced control upon treatment with ENZ (19% for 20 uM and 30% for 40 uM) indicating a synergistic effect of miR-299-3p and ENZ (Supplementary data Fig. S3D) Furthermore, cell viability was reduced by 10-20% in the miR-299-3p expressing LNCaP-104S cells with and without ENZ treatment (Supplementary data Fig. S3D). These results indicate that miR-299-3p expression is potentially contributing to improved ENZ and DTX sensitivity of CRPC cells.

### Expression of miR-299-3p delays tumor growth in xenograft models

**Figure 3:**
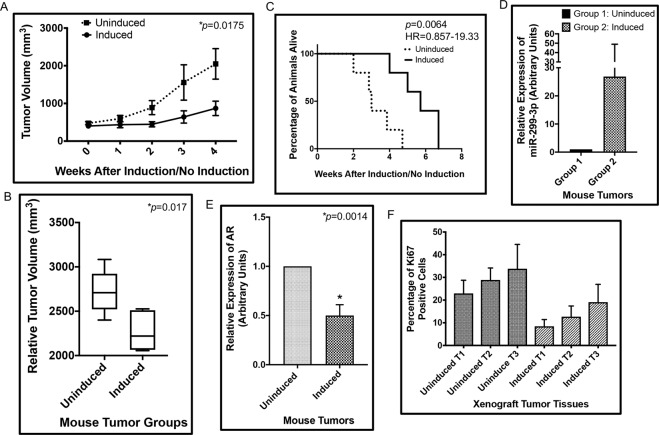
Expression of miR-299 reduced tumor growth in xenograft models. C4-2B-299 cells were used to establish tumors in the flank of NSG mice. (**A**) Progression of tumor growth upon induction or no induction of miR-299 expression in the tumors. Mice were injected with C4-2B-299 cells and induced with feed containing doxycycline (dox-feed) or regular feed. Tumor growth was monitored for 28 days post induction by tumor volume measurement. Mice receiving the dox-feed showed a slower tumor growth compared to the control group of mice. (**B**) Average tumor volume showing a significant reduction in overall tumor volume in mice received dox-feed compared to the mice received regular feed at the time of euthanasia. Data show mean ± SD of tumor volumes of 5mice/group. (**C**) Mice receiving the dox-feed also were alive for a significantly longer time based on the time elapsed to reach the specified 1.5 cm^3^ tumor volume required for euthanization compared to the uninduced control group. (**D,E**) Quantitative RT-PCR showing increased expression of miR-299-3p (**D**) and reduced expression of AR (**E)** in tumors from mice that received dox-feed compared to the regular feed. Data show mean ± SD of three tumors from each group. (**F**) Quantification of Ki67^+^ cells in tumors from mice that received dox-feed compared to the regular feed. Data show percentage of Ki67^+^ cells in three tumors from each group.

Our previous experiments showed loss of expression of miR-299-3p in prostate tumor tissues and anti-proliferative effects of miR-299-3p replenishment in CRPC and metastatic PCa cells. In this study, we examined the antitumor effects of miR-299-3p expression in xenograft models *in vivo*. We injected inducible C4-2B-299 cells into the flanks of NSG mice and monitored tumor growth with or without doxycycline treatment for 28 days after starting induction. We compared the tumor volumes up to 4 weeks as all animals in the uninduced group were euthanized just past 4 weeks because of the increased tumor burden, whereas animals receiving doxycycline had slow growing tumors and were maintained for a longer period of time until they were euthanized when tumors reached 1.5 cm^3^ in volume. Tumor volume analysis showed a significantly reduced tumor growth (*p* = 0.029) at 4 weeks in the doxycycline treated group compared to the group without doxycycline treatment (Fig. 3A). Overall, harvested tumors of the doxycycline treated group were significantly smaller (*p* = 0.017) than from the uninduced group (Fig. 3B). The doxycycline treated group of animals also showed a significantly longer elapsed time before euthanization (*p* = 0.006) compared to the uninduced group (Fig. 3C). Analysis of tumor tissues showed a higher expression of miR-299-3p (Fig. 3D), and reduced expression of AR (Fig. 3E) and Ki67 proliferation marker (Fig. 3F and Supplemental data Fig. S4) in the doxycycline treated groups compared to the uninduced group. This observation confirms, *in vivo*, the contribution of miR-299-3p to antitumor effects noted in CRPC cells *in vitro*.

### Expression of miR-299-3p reduces expression of AR, AR-v7 and VEGFA and facilitates association of AR and VEGFA mRNA in RNA- induced silencing complex (RISC) of CRPC cells

**Figure 4:**
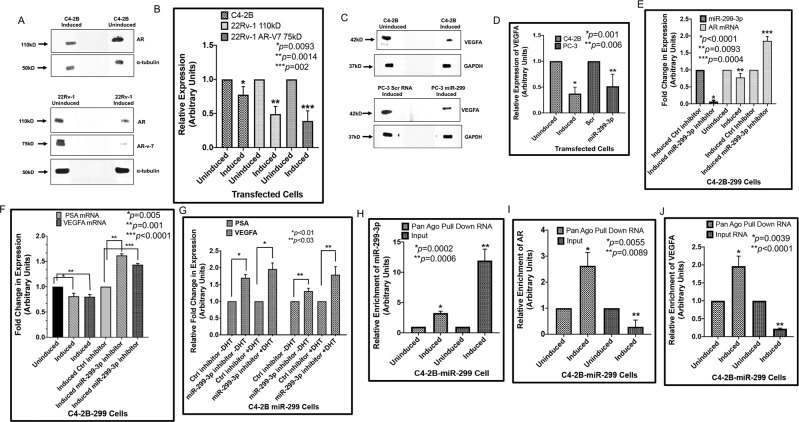
miR-299-3p expression reduced expression of AR, PSA and VEGFA, and increased miRNA/mRNA interaction in RISC. All data show the mean ± SD of at least 3 independent experiments. (**A**) Immunoblot analysis showing reduced expression of full length AR and AR-V7 in lysates from induced C4-2B-299 and 22Rv-1-299 cells overexpressing miR-299-3p compared to uninduced cells. Alpha tubulin was used as the internal control. (**B**) Densitometric analysis of full-length AR and AR-v-7 protein expression normalized to internal controls. (**C**) Immunoblot analysis showing reduced expression of VEGFA in lysates of induced C4-2B-299 and PC-3-299 cells compared to uninduced or induced Scr RNA expressing PC-3 cells. GAPDH and α-tubulin were used as the internal controls. (**D**) Densitometric analysis of VEGFA protein expression normalized to internal controls. (**E,F**) Quantitative RT-PCR of miR-299-3p, AR, PSA and VEGFA in RNA extracted from the induced C4-2B-299 cells transfected with miR-299-3p inhibitor or control inhibitor. (**E**) Average fold-change in expression of miR-299-3p and AR showing reduced expression of miR-299-3p and increased expression of AR mRNA in induced C4-2B-299 cells upon transfection of miR-299-3p inhibitor compared to the control inhibitor. Decreased expression of AR mRNA could be noted in the untransfected induced C4-2B-299 cells compared to the uninduced cells. (**F**) Average fold-change in expression of PSA and VEGFA mRNAs showing rescue effect upon transfection of miRNA inhibitor compared to the control inhibitor. Reduced expression of PSA and VEGFA mRNAs could be noted in the untransfected but induced C4-2B-299 cells. (**G**) Quantitative RT-PCR of PSA and VEGFA in RNA extracted from the induced C4-2B-299 cells transfected with miR-299-3p inhibitor or control inhibitor followed by treatment with and without dihydrotestosterone (DHT). Average fold change in expression showing increased expression of PSA and VEGFA mRNAs in the absence of DHT treatment in cells transfected with miR-299-3pi. Treatment with DHT showed greater increase in PSA and VEGFA mRNA expressions in miR-299-3pi transfected cells compared to control inhibitor. (**H**–**J**) RNA-immunoprecipitation studies with the pan-AGO antibody was used in the pulldown of interacting miRNAs-mRNAs complexes. Total RNA extraction from pulldown samples followed by qRT-PCR analysis revealed enrichment of miR-299-3p (3.3-fold), AR mRNA (2.6-fold) and VEGFA mRNA (1.96-fold) in the induced C4-2B stable line expressing miR-299-3p compared to the uninduced control. Quantitative RT-PCR of miR-299-3p, AR and VEGFA in the input RNA from induced C4-2B-299 cells showing increased miR-299-3p, and decreased AR and VEGFA mRNAs compared to uninduced cells.

Previously, luciferase reporter assays have shown that AR mRNA is targeted by miR-299-3p in PCa cells and reduces AR and AR-V7 in LNCaP and 22Rv-1 PCa cells^19^. In support of this finding, C4-2B-299 and 22Rv-1-299 cells showed reduced expression of AR and AR-V7 proteins and AR mRNA upon miR-299-3p induction by doxycycline treatment compared to uninduced conditions (Fig. 4A,B and Supplementary data Fig. S5A,B). Reduced expression of PSA mRNA as a transcriptional target of AR, was also noted in both inducible stable sublines upon induction of miR-299-3p expression (Fig. S5A,B). In silico analysis showed that VEGFA is another target of miR-299-3p. Because VEGFA is transcriptionally regulated by AR through binding to the VEGFA promoter in a complex with Sp1^24^, we studied the expression of VEGFA in these cells. We noted a significant reduction of VEGFA proteins (2-13-fold) and mRNA (12–25%) in both C4-2B-299 and 22Rv-1-299 cell lines as well as in PC-3 cells expressing miR-299-3p upon induction. No reduction could be noted in uninduced cells or induced control RNA expressing PC-3 cells (Fig. 4C,D,F and Supplementary data Fig. S5C–E).

The effect of miR-299-3p on AR, PSA and VEGFA was further confirmed by monitoring the rescue effect of miR-299-3p inhibitor (miR-299-3pi). Induced C4-2B-299 cells when transfected with miR-299-3pi or control inhibitor showed significant restoration of AR, PSA and VEGFA mRNA expression in miR-299-3pi transfected cells (Fig. 4E,F). We also noted a significant increase in AR and VEGFA protein expression in these cells (Supplementary data Fig. 5F,G). Furthermore, induced 22Rv-299 cells when transfected with miR-299-3pi showed significant reversal in expression of full length AR (5.7-fold) and AR-v-7 (5.8-fold) protein expression compared to control inhibitor transfected cells (Supplementary data Fig. S5H,I). To confirm the activity of AR in miR-299-3pi treated C4-2B cells, we examined the effect of DHT on the induced cells transfected with miR-299-3pi. Our results showed an increased expression of PSA (70%) and VEGFA (30%) in DMSO treated cells which was further increased to 2-fold (PSA) and 80% (VEGFA) compared to the control cells (Fig. 4G). This observation indicates that the AR is functionally active in miR-299-3p depleted C4-2B cells.

**Figure 5:**
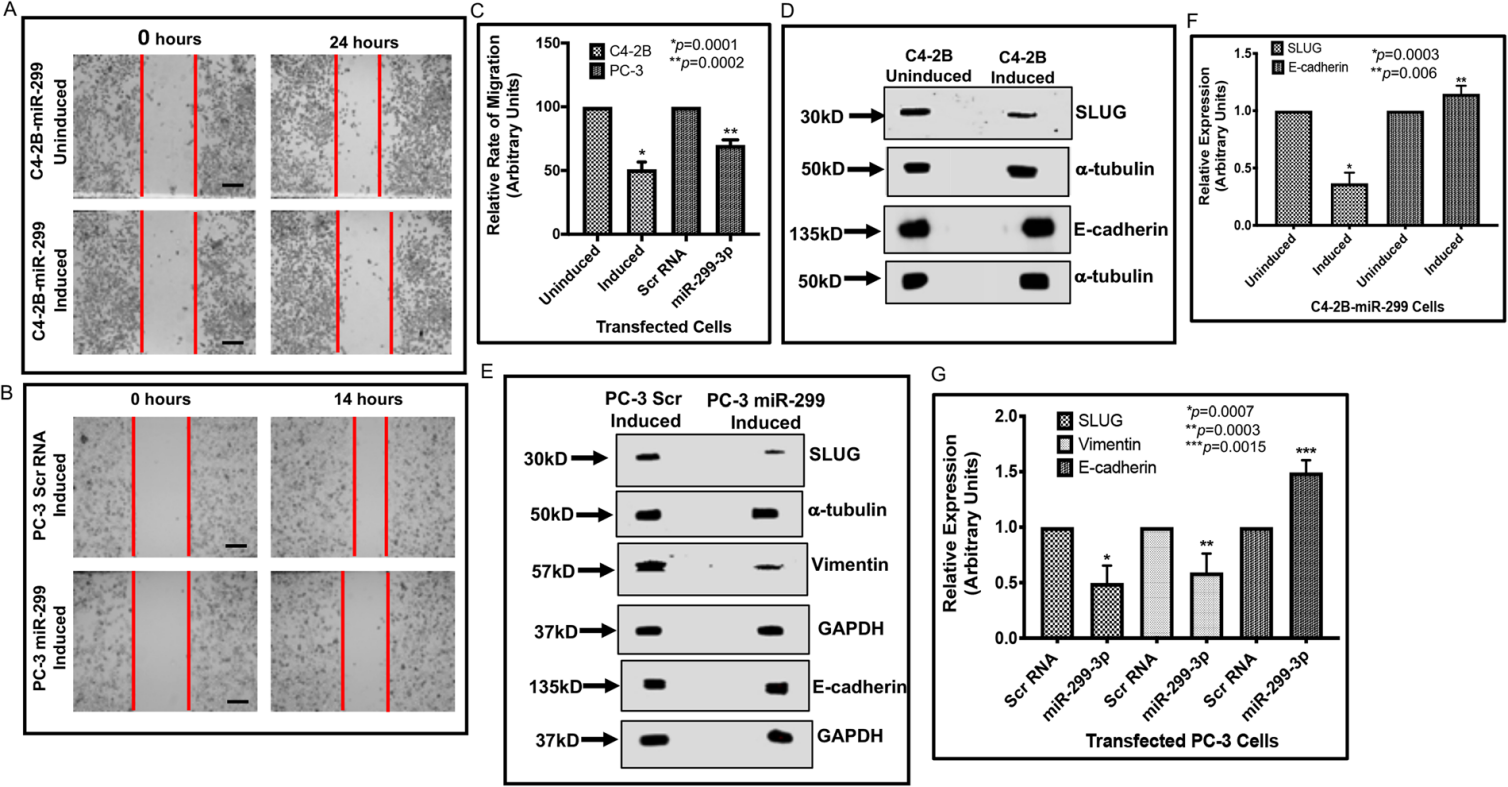
Expression of miR-299 decreased cell migration and promoted an epithelial phenotype. Migration and expression of EMT markers were compared between induced and uninduced C4-2B cells stably expressing miR-299-3p, and between induced PC-3 cells transiently expressing miR-299-3p or scr RNA. (**A,B**) Bright field images taken at 0 hours and 24 hours (C4-2B) or at 0 hours and 14 hours (PC-3) after making the scratch showing the relative rates of migration of C4-2B-299 induced cells compared to the uninduced cells and induced PC-3-299 cells compared to the induced scr RNA expressing cells. Two scratches were made in each dish and the experiments conducted in triplicate. Scale bar: 200 μm. (**C)** Relative rates of cell migration. Data presented as the ratio of the distance migrated by the uninduced or induced scr RNA control cells compared to induced cells expressing miR-299. Data show the mean ± SD of minimum three independent assays. (**D**–**G**) Expression of miR-299 promoted conversion of EMT to epithelial phenotype. (**D,E**) Representative images of the immunoblot analysis of Slug, vimentin (PC-3 cells) and E-cadherin in induced and uninduced C4-2B-299 cells and induced PC-3-299 or scr RNA control PC-3 cells. Antibodies against α-tubulin and GAPDH were used as the loading controls. (**F,G**) Densitometric analysis showing reduced expression of Slug and vimentin, and increased expression of E-cadherin in induced cells compared to the uninduced or induced scrRNA control cells. Values were normalized to internal controls. Data represent mean ± SD of at least 3 independent assays.

However, all these findings provide only indirect evidence for miR-299-3p regulating AR and VEGFA. A more direct association of AR and VEGFA has been implicated using AGO-RISC (Argonaut RNA-induced silencing complex) immunoprecipitation experiments. We studied the enrichment of miR-299-3p, AR and VEGFA mRNAs in RISCs by AGO pull-down assays in cells overexpressing miR-299. Our results showed significant enrichment of miR-299-3p (3.2-fold), and AR (2.6-fold) and VEGFA (2.0-fold) mRNAs in RISCs of induced C4-2B-299-3p cells compared to uninduced cells (Fig. 4H–J). Input RNA from induced and uninduced cells showed comparable expression pattern of increased miR-299-3p and decreased AR and VEGFA mRNAs upon induction (Fig. 4H–J). These observations provide further evidence that overexpression of miR-299-3p favors association of AR and VEGFA in RISCs.

### Expression of miR-299-3p reduces cell migration and alters expression of epithelial-mesenchymal transition (EMT) markers

An important feature of many aggressive cancer cells is the increased motility. We used induced C4-2B-299, 22Rv-1-299 and PC-3-299 as CRPC cell models to test cell migration and expression of mesenchymal markers such as Slug^25^, vimentin^26^, and the epithelial marker, E-cadherin^27^. The scratch assay images and quantification of the distance traversed by the cells at 14 hr (PC-3) and 24 hr (C4-2B and 22Rv-1) showed significant inhibition (26–49%) of migration of PC-3 (49%), C4-2B (30%) and 22Rv-1 (26%) cells upon miR-299-3p overexpression (Fig. 5A–C, and Supplementary data Fig. S6A,B) compared to uninduced cells or induced PC-3 cells expressing Scr RNA. These results were supported by immunoblot assays showing reduced expression of Slug, and vimentin but increased expression of E-cadherin (Fig. 5D–G and Supplementary Fig. S6C,D).

### miR-299-3p mediates inhibition of VEGFA is associated with altered activation of Slug possibly through PI3K/Akt pathway

**Figure 6:**
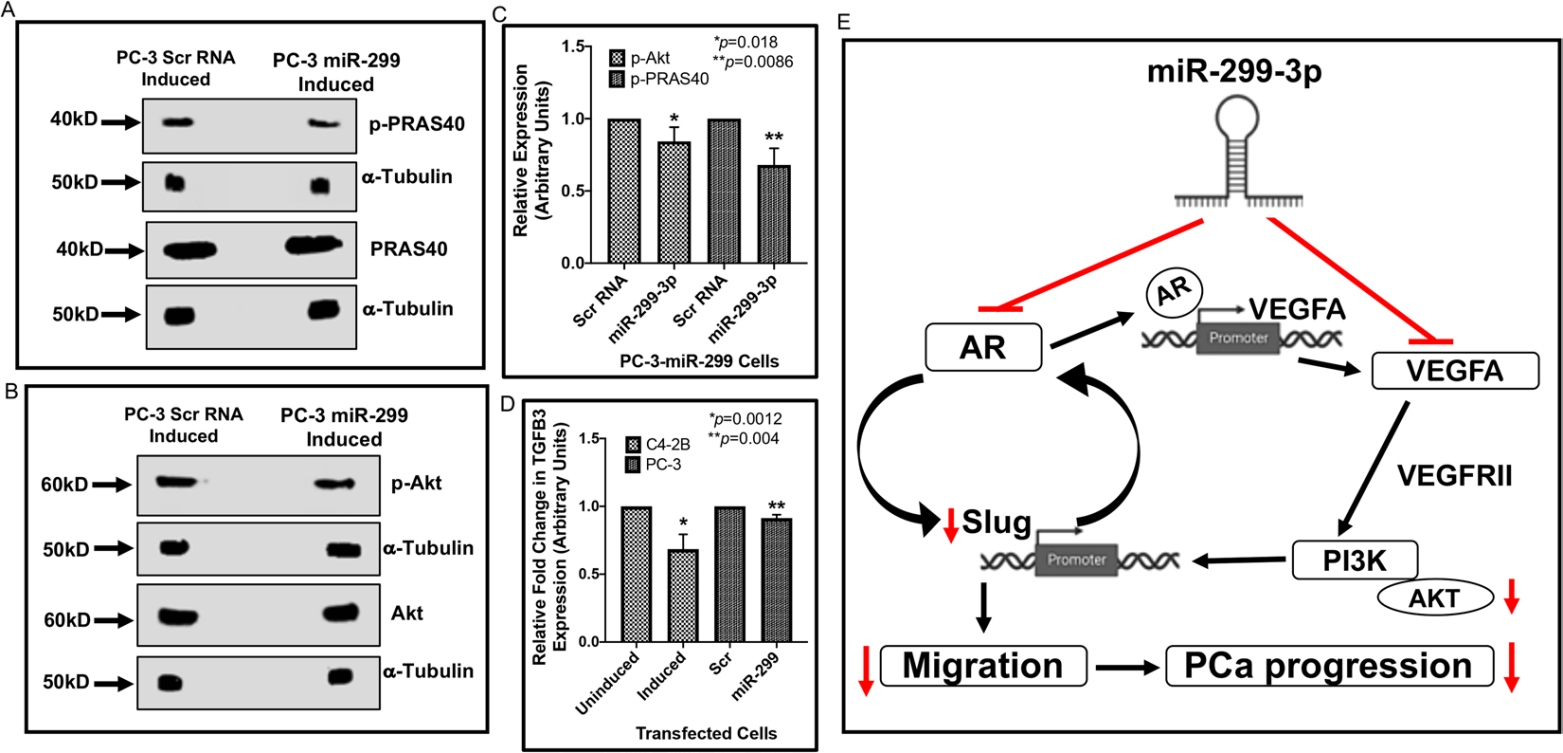
Expression of miR-299-3p repressed activation of the PI3K/Akt pathway and downstream effector of Slug. Immunoblot analysis of phospho-Akt and phospho-PRAS40 (Akt substrate) expression and qRT-PCR of TGFB3 mRNA were performed in C4-2B and PC-3 cells overexpressing miR-299-3p cells. (**A,B**) Representative images of the immunoblots of Akt, pAkt, PRAS40 and pPRAS40 in the lysates of induced PC-3-299 and scr RNA control PC-3 cells. Antibodies against α-tubulin was used the internal control. (**C**) Comparative analysis of Akt and PRAS40 expression showing significant reduction in expression of the phosphorylated Akt and PRAS40 in PC-3-299 cells expressing miR-299-3p. Values were normalized with the internal control. Data show the mean ± SD of at least three independent assays. (**D**) Quantitative RT-PCR showing decreased TGFB3 mRNA expression in induced C4-2B-299 and PC-3-299 cells expressing miR-299-3p compared to uninduced or control PC-3 cells. Data show the mean ± SD of at least three independent assays. (**E**) A model showing miR-299-3p mediated regulation of AR and VEGFA and inactivation of downstream effectors Slug and PI3K/Akt leading to inhibition of migration and PCa progression.

Analysis of VEGFA expression showed significant downregulation of VEGFA in PC-3 cells expressing miR-299-3p compared to the control cells. VEGFA is one of the key proteins that promote metastatic PCa and activation of Slug through the PI3K/Akt pathway in an autocrine manner^28,29^. As VEGFR2 binding by VEGFA has been implicated in enhancing the migration potential of PC-3 cells^30^, we examined the expression and activation of Akt and Akt substrate PRAS40 by Western blot analysis to determine the signaling pathway affected by reduced expression of VEGFA. Our results showed significant downregulation of pAkt (20%) and pPRAS40 (32%) in PC-3 cells overexpressing miR-299-3p compared to control cells although total Akt and PRAS40 levels remained unchanged (Fig. 6A–C). To examine inhibition of EMT as a result of inactivation of Akt signaling, we monitored expression of the TGFB3 mRNA, a downstream effector of Slug in transfected sublines. TGFB3 is produced as a result of inhibition of E-cadherin by Slug^25^ and promotes EMT. Our results showed 10–33% reduction in expression of TGFB3 mRNA in induced C4-2B-299, PC-3-299 and 22Rv-1-299 cells compared to uninduced or induced control PC-3 cells (Fig. 6D and Supplementary data Fig. S6E). These observations demonstrate inhibition of E-cadherin function that facilitates epithelial-mesenchymal transition (EMT). Based on these observations, we proposed a model of the function of miR-299-3p that is lost in PCa (Fig. 6E)

## Discussion

In this study, we investigated the tumor suppressor function of miR-299-3p. Our expression analysis using prostate tumors and the TCGA database revealed a negative association of miR-299-3p with PCa. The expression pattern of miR-299-3p in tumor tissues is corroborated with its low expression in PCa cells. Because miRNA functions are diverse and context dependent, we performed an analysis of miR-299-3p functions using PCa cells of different aggressiveness and AR status. We used three CRPC cells which express full-length AR (C4-2B), full-length AR, and the spliced variant AR-V7 (22Rv-1), and nonfunctional AR (PC-3). To our knowledge, this is the first report of expression and functional characterization of miR-299-3p in PCa.

Our expression analysis result is in support of previous studies showing association of lower miR-299-3p expression with poor prognosis and worse survival of hepatocellular carcinoma patients^14^. Similarly, a negative correlation with miR-299-3p expression and colorectal cancer has been reported^15^. However, an opposite expression pattern, thus overexpression of miR-299-3p in ovarian cancer has been reported^31^. This disparate expression pattern of miR-299-3p in different cancers further affirms that miRNA functions are likely tissue and cellular environment specific. To understand the functional specificity of miR-299-3p in PCa, we used an approach of controlled replenishment and depletion of miR-299-3p in different PCa cells. Our results provide evidence of contribution of miR-299-3p as an antiproliferative, antitumor, proapoptotic, and antimigratory factor in PCa cells irrespective of their AR status. Our analysis of the underlying mechanism of cell cycle control revealed two different modes of inhibition, one in G1/S and the one in G2/M phase in AR-positive and AR-negative cells through inhibition of CCND1 and CCNB1 expression, respectively. Our study also showed the potential benefit of miR-299-3p replenishment on improving drug sensitivity. Expression of miR-299-3p led to increased sensitivity of C4-2B and 22Rv-1 cells to ENZ and DTX and PC-3 cells to DTX. An androgen dependent cell line LNCaP-104S transfected with miR-299-3p also showed improved drug sensitivity to ENZ demonstrating a synergistic effect of miR-299-3p and ENZ, which confirms that the effect is not cell line specific. Transfection with miR-299-3p mimic also showed improved sensitivity of lung cancer cells to doxorubicin^16^. The effect of miR-299-3p replenishment on antiproliferative effects was further confirmed *in vivo* showing decreased tumor volume and tumor progression, and reduced AR and Ki67 expression. However, we cannot rule out the possibility of contributions of miR-299-5p to the phenotypic changes and antitumor effects noted in PCa cells upon doxycycline treatment. A previous study reported that, expression of miR-299-5p mimic showed inhibition of migration in AR-independent PC-3 and DU145 PCa cells, and increased apoptosis in DU145 cells but not in PC-3 cells^13^. On the other hand, miR-299-5p did not show any antiproliferative and antitumorigenic effects in this study. On the contrary, a tumor promoter role of miR-299-5p has been noted in hepatocellular carcinoma and glioblastoma^32,33^. This information strengthens our interpretation that the tumor suppressor effects noted in miR-299 expressing cells is, to the most part, the result of miR-299-3p functions.

Our study has outlined the mechanistic aspect of the miR-299-3p induced antimigratory phenotype of both AR-positive and AR-negative PCa cells showing reduced expression of Slug. Additionally, overexpression of miR-299-3p altered expression of different Slug-target genes such as *E-cadherin* and *TGFB3*. Slug belongs to the Snail/Slug superfamily and is known to be involved in epithelial-mesenchymal transition and metastasis in different cancers^34^, and overexpression of Slug is noted in CRPC tissues^35^. However, predicted binding sites of miR-299-3p or -299-5p in the 3′UTR of Slug were not identified, which suggests that miR-299 has an indirect effect on Slug expression in PCa through modulation of other target genes.

Our study showed decreased expression of AR and VEGFA upon expression of miR-299-3p, and increased number of AR and VEGFA mRNAs and miR-299-3p in RISCs of miR-299 overexpressing cells. These results were supported by published studies using luciferase reporter assays that showed that AR and VEGFA can be directly targeted by miR-299-3p^15,19^. Our study also showed that knockdown of miR-299-3p in the stable C4-2B cell line expressing miR-299-3p restored AR and VEGFA expression. Additionally, DHT treatment of induced C4-2B cells transfected with miR-299-3p inhibitor revealed increased expression of AR-target gene PSA and VEGFA compared to the control which further highlights the bimodal effect of miR-299-3p in regulation of AR activity and VEGFA. However, inhibition of miR-299-3p and ENZ treatment did not show an effect on VEGFA expression although a significant decrease in PSA expression was observed (Data not shown). These data suggest that miR-299-3p and ENZ have a stronger effect in inhibiting AR activity and the effect on VEGFA expression is potentially mediated through a direct effect of miR-299-3p in an AR-independent pathway. Studies have shown that VEGFA/VEGFR2 is an important signaling axis in CRPC which allows the cancer cells to survive, proliferate and metastasize in an AR-independent manner^36,37^. Treatment of DTX in miR-299-3p expressing AR-negative PC-3 cells showed further reduction in expression of VEGFA compared to control cells as well as DMSO control (Data not shown). These results suggest that miR-299-3p is involved in regulation of VEGFA and promotes an antiangiogenic environment in PCa cells during combination treatment with DTX, which can have a potential impact in treatment of AR-independent CRPC.

Based on our results it could be surmised that altered expression of AR and VEGFA could impact Slug expression and contribute to overall PCa progression. A previous study has shown that constitutively active AR can drive Slug expression which further enhances AR expression and activity through a synergistic feedback effect. This relationship between AR and Slug signaling allows for the growth and survival of PCa cells, specifically in CRPC that has a constitutively active AR^35^. Studies have also shown that VEGFA in an unique androgen-regulated gene with AR involved in the transcriptional activation of VEGFA, which may further play an autocrine role in promoting PCa progression^38^. Multiple studies have also shown the involvement of VEGFA in promoting EMT by inducing expression of Snail and Snail family members^39^. Taken together, we propose a model that describes the bimodal action of miR-299-3p as a tumor suppressor in PCa by targeting both AR and VEGFA simultaneously.

In the AR-positive cells, it can be assumed that AR signaling is the dominant pathway that drives cell proliferation and migration through various AR target genes such as VEGFA and Slug (Fig. 6E). However, in the aggressive AR-negative CRPC cells, upregulation of VEGFA could drive expression of Slug through the VEGFA/VEGFR2 autocrine signaling axis in an AR-independent manner, which promotes cancer cell survival and positively influence PCa progression. To determine the functional relationship between VEGFA and Slug in PCa, we focused on the PI3K/Akt pathway which is a common downstream signaling outcome of activation of the VEGFA/VEGFR2 signaling axis and which plays an important role in cancer cell survival^28^. Multiple studies showed that the PI3K/Akt pathway can directly contribute to EMT with a positive correlation between phosphorylated Akt expression and Slug expression^29^. Furthermore, PI3K/Akt induced degradation of GSK-3B involved in ubiquitination and proteolysis further stabilizes Slug expression^40^. In the AR-negative PC-3 cells, we showed that downregulation of VEGFA upon overexpression of miR-299-3p was associated with reduced expression of Slug and reduced expression of phosphorylated Akt and its substrate, phosphorylated PRAS-40 although the levels of total Akt and PRAS-40 remained unchanged. These observations suggest that changes in VEGFA expression alter the PI3K/Akt signaling pathway, which is commonly activated in CRPC^41^. Based on our data, we speculate that VEGFA/VEGFR2 autocrine mediated activation of Slug is primarily through PI3K/Akt signaling which further promotes PCa progression and survival.

Targeting AR, VEGFA and PI3K/Akt signaling pathways individually are some of the common targeted therapies used in PCa^28,42,43^, however, resistance to these therapies eventually develops. We speculate that a combination approach may be used to overcome development of resistance to a single targeted therapy. MicroRNAs may be important tools to aid in this combination therapy. Our study shows that miR-299-3p targets both AR and VEGFA and could impact multiple pathways involved in cell proliferation, cell survival and EMT simultaneously in both AR-positive and AR-negative PCa cells. Additionally, miR-299-3p overexpression contributed to delayed tumor progression *in vivo* suggesting an overall tumor suppressor effect in PCa. Thus, our study demonstrates a novel application of miR-299-3p as a multifaceted therapeutic target in prostate cancer.

## Materials and Methods

### Acquisition of patient tissues

Prostate tissues were obtained from Cooperative Human Tissue Network (Southern division) collected at the University of Alabama at Birmingham (UAB) under Institutional Review Board (IRB) approved protocol and after obtaining informed consent from donors for this type of research. Additionally, the study using deidentified human tissues has been granted an exemption from ethics approval by the University of Central Florida IRB. Formalin-fixed paraffin embedded prostate tissues were macro-dissected separately for tumor areas and uninvolved areas at UAB and used for RNA extraction followed by qRT-PCR for the miR-299-3p as described previously^12^. TCGA PANCAN data was used for analysis. The miRNA data was extracted from “pancanMiRs_EBadjOnProtocolPlatformWithoutRepsWithUnCorrect MiRs_08_04_16.txt” available from https://gdc.cancer.gov/about-data/publications/pancanatlas. Values below zero were set to zero and all values were log_2_ transformed, log_2_(x + 1). The 333 tumor samples from the TCGA PRAD publication^44^ were extracted together with the Normal Prostate samples resulting in 330 matching tumor and 51 normal samples.

### Cell lines and transfection

AR-positive LNCaP-104S (a gift from Dr. Shutsung Liao, University of Chicago), MDA-PCa-2b (ATCC), 22Rv-1 (ATCC) and C4-2B (a gift from Dr. Leland Chung, Cedars-Sinai Medical Center) cells, AR-negative PC-3 (ATCC) cells and non-tumorigenic RWPE-1 (ATCC) cells were used for various experiments. C4-2B and 22Rv-1 cells were transfected with pLVX-TetOne-miR-299 using Lipofectamine 3000 transfection reagent (ThermoFisher Scientific) and selected for stable sublines using puromycin. Next, cells were induced with doxycycline (1 μg/mL) and ZsGreen positive cells were sorted to generate C4-2B-299 and 22Rv-1-299 stable lines. For all experiments, doxycycline-induced C4-2B-299 and 22Rv-1-299 cells were used along with uninduced stable lines serving as the controls. PC-3 and LNCaP-104S cells were transiently transfected with the SMARTvector inducible lentiviral shRNA (Dharmacon) containing the cDNAs for scrambled RNA or pLVX-TetOne-miR-299 and used in subsequent experiments.

For knockdown studies, C4-2B-299 and 22Rv-299 cells were induced with doxycycline and expression of miR-299 was confirmed by microscopy of fluorescent cells. Cells were transfected with miR-299-3p inhibitor (miRIDIAN microRNA hairpin inhibitor, Dharmacon, MIMAT0000687) or negative control siRNA (miRIDIAN negative control hairpin, Dharmacon) using RNAiMax (Thermo Fisher Scientific). All knockdown experiments were performed 48 hours after transfection.

### Cell proliferation and dual label immunofluorescence assay

C4-2B-299 and 22Rv-1-299 sublines and PC-3 cells expressing scrambled RNA or miR-299 were induced with doxycycline for 48 hr and cell proliferation assays were conducted using MTS based CellTiter Aqueous One solution cell proliferation assay kit (Promega). For immunofluorescence analysis, cells were seeded on poly-l-lysine coated coverslips and induced with doxycycline for 72 hours. Cells were fixed with 4% paraformaldehyde and probed with anti-Ki67 primary antibodies (Cell Signaling) followed by Cy3-conjugated goat anti-mouse secondary antibodies (Vector Laboratories). Cells were visualized using a Leica SP5 confocal inverted microscope equipped with Leica LAS AF software to analyze positive signals. ImageJ software was used to quantify the number of Ki67 positive cells in each field.

### Cell cycle, apoptosis and drug sensitivity assay

C4-2B-299 and 22Rv-1-299 sublines and transfected PC-3 cells expressing miR-299 or scrambled RNA were induced with doxycycline and processed for cell cycle analysis using Propidium iodide staining. For apoptosis assays, induced and uninduced C4-2B-299 and 22Rv-1-299 cells were used for annexin V staining using a PE Annexin V Apoptosis Detection Kit (BD Biosciences) as per manufacturer’s protocol. For drug sensitivity experiments, C4-2B-299, 22Rv-1-299, PC-3 and LNCaP-104S cells were seeded in charcoal-stripped FBS (CS-FBS) containing growth media. Twenty-four hours post seeding, expression of miR-299 was induced with doxycycline (1 µg/mL) or cells were left uninduced as controls for C4-2B-299 and 22Rv-299 stable lines. For LNCaP-104S and PC-3 cells, 24 hours post seeding, cells were transfected with the SMARTvector inducible lentiviral shRNA (Dharmacon) containing the cDNAs for scrambled RNA or pLVX-TetOne-miR-299 followed by doxycycline induction 24 hours post transfection. Cells were treated with different concentrations of Enzalutamide (ENZ) or Docetaxel (DTX) for 72 hr before cell viability was analyzed using the MTS-based Cell Titer Aqueous One solution cell proliferation assay kit (Promega) as per manufacturer’s protocol.

### RNA immunoprecipitation assay

RNA immunoprecipitation (RIP) was performed using the EZ-Magna RIP RNA Binding Protein Immunoprecipitation kit (EMD Millipore) as per manufacturer’s protocol. Uninduced and induced C4-2B-299 cells at 48 hours post induction with doxycycline were lysed in RIP lysis buffer followed by incubation with magnetic beads conjugated to Anti-Pan Ago mouse monoclonal antibodies (EMD Millipore) or mouse IgG negative control antibodies (EMD Millipore). Following immunoprecipitation, total RNA was isolated by phenol-chloroform extraction and ethanol precipitation. Pulled-down RNA was quantified and evaluated using an RNA agarose gel. Input RNA and pulled down RNA were used for cDNA synthesis and qRT-PCR for miR-299-3p, AR mRNA and VEGFA mRNA quantifications.

### Scratch assay

C4-2B-299 and 22Rv-1-299 stable cells and PC-3 cells expressing scrambled RNA or miR-299 were used for scratch assays. Cells were induced with doxycycline and scratches were made. Migration of cells within the scratch was visualized and distance traversed quantified at 0 and 14 hrs for PC-3 cells and at 0 and 24hrs for C4-2B-299 and 22Rv-1-299 cells by microscopy.

### Xenograft model for tumorigenicity assay

Xenograft experiments were performed using 6-8 weeks old NSG (NOD.Cg-*Prkdcscid*Il2rg*tm1Wjl*/SzJ (005557)) mice (Jackson Laboratory) maintained under pathogen-free conditions. Xenograft experiments were performed according to relevant guidelines and regulations using an animal protocol approved by the Animal Ethics Committee/Institutional Animal Care and Use Committee of the University of Central Florida.

C4-2B-299 cells (6 × 10^6^ cells/mouse) mixed with 0.1% matrigel in 100 μl volume were injected subcutaneously into the flank. Once tumor volume reached 300 mm^3^, animals were randomly selected for uninduced group (5 mice/group) receiving regular feed and induced groups receiving Dox-feed (625 mg doxycycline/kg, Envigo Teklad Diets). Tumor growth was monitored using a caliper and tumor volume was calculated as 0.52 × length × height × width as the tumors grew. Tumors were harvested after the specified time and tumor tissue architecture examined by H&E staining of formalin fixed paraffin embedded sections. Tumors were used for RNA extraction followed by quantitative real-time PCR (qRT-PCR) analysis of miR-299-3p and AR mRNA. Expression of Ki67 proliferation marker was detected using immunofluorescence staining.

### Statistical analysis

All statistical analyses were performed using Student’s t-test or one-way ANOVA for independent measures using GraphPad Prizm. The statistical significance was set at *p* < 0.05.

A detailed description of all the methods, including total RNA extraction, qRT-PCR, maintenance of the PCa cell lines used, protein extraction, western blotting, cell cycle, apoptosis, scratch assay and immunohistochemistry are presented in the supplementary data.

### Ethics approval and consent to participate

Ethics approval and consent to participate was obtained from Cooperative Human Tissue Network (Southern division) at the University of Alabama at Birmingham (UAB) in accordance with an approved IRB protocol.

## Supplementary information

Supplementary Information.

## Data Availability

The datasets used in this manuscript and all analyzed data from this study are available from the corresponding author upon reasonable request.
