# Supplementary material for: Multifaceted Function of MicroRNA-299-3p Fosters an Antitumor Environment Through Modulation of Androgen Receptor and VEGFA Signaling Pathways in Prostate Cancer
Source: Sci Rep. 2020 Mar 20;10:5167. doi: 10.1038/s41598-020-62038-3 (PMC7083835; doi:10.1038/s41598-020-62038-3)
Supplement: Supplementary file 1 — Supplementary Information. [file 41598_2020_62038_MOESM1_ESM.pdf]

Multifaceted Function of MicroRNA-299-3p Fosters an Antitumor Environment Through Modulation of Androgen Receptor and VEGFA Signaling Pathways in Prostate Cancer.

Kavya Ganapathy[1], Stephen Staklinski[1], Md Faqrul Hasan[1], Richard Ottman[1], Thomas Andl[1], Anders Berglund[2], Jong, Y. Park[3] and Ratna Chakrabarti[1]*

Supplementary Data

Methods:

RNA extraction and qPCR: Total RNA were extracted from prostate tissues or cancer cell lines using RecoverAll (ThermoFisher) or the Direct-zol RNA extraction kit (Zymo Research). cDNA synthesis was performed using the QuantiMir RT Kit (System Biosciences) or the miScript RT II Kit (Qiagen). Expression of miR-299-3p was determined by quantitative real-time-PCR (qRT-PCR) using specific primers for miR-299-3p and 3 internal snRNAs (System Biosciences) and SYBR Green PCR reagents (ThermoFisher). For mRNAs, specific primers for the target genes and EIF3D and RPL13A as the internal controls (Quantitect primers, Qiagen), miScript SYBR green PCR kit (Qiagen) and Power-UP SYBR Green (Thermo Fisher Scientific) or Quantitect SYBR Green master mix (Qiagen) were used. qRT-PCR of miRNA/mRNAs were performed on the QuantStudio 7 thermal cycler from Applied Biosystems and data analyzed using SDS2.3 software (ABI). DNA concentrations were analyzed based on SYBR Green fluorescence normalized to the passive reference dye, ROX. The obtained Ct values were normalized to the internal controls and Ct values were used to derive the 2ΔΔCt values using the miRNome analysis software (SBI).

Cell line maintenance:

All experiments with cell lines were performed within one month after thawing of cryopreserved cells. All cells are rigorously monitored for mycoplasma contamination using DAPI staining method and all cell lines were authenticated through STR profiling.

AR positive 22Rv-1 (ATCC) and C4-2B (a gift from Dr. Leland Chung, Cedars-Sinai Medical Center) cells were maintained in RPMI-1640 medium (Sigma Aldrich) supplemented with 10% FBS and 1% antibiotic/antimycotic (Invitrogen). AR negative PC-3 (ATCC) cells were maintained in F-12K HAM media (Sigma Aldrich) containing 10% FBS and 1% antibiotic/antimycotic. LNCaP-104S cells (a gift from Dr. Shutsung Liao, University of Chicago) were maintained in DMEM (Sigma Aldrich) containing 10% FBS, 1% antibiotic/antimycotic and 1 nM DHT (Sigma Aldrich). MDA-PCa-2b (ATCC) cells were

maintained in 10% F-12K HAM medium containing 10% not heat inactivated FBS, 1% antibiotic/antimycotic, 25ng/mL cholera toxin, 10ng/mL mouse EGF, 0.005 mM phosphoethanolamine, 100 pg/mL hydrocortisone, 45nM sodium selenite, 0.005 mg/mL insulin. Non-tumorigenic RWPE-1 (ATCC) cells were maintained in Keratinocyte Serum Free Medium (Gibco) supplemented with 0.05 mg/mL BPE and 5ng/mL EGF.

Preparation of inducible pLVX-TetOne-miR-299 construct:

For ectopic expression, a doxycycline-inducible construct encompassing human miR-299 precursor sequence obtained from OriGene (MI0000744) was prepared and cloned into the pLVX-TetOne-Puro vector (Clontech) at the BamH1 site, harboring a ZsGreen fluorescent marker between BamHI and EcoRI restriction sites.

Cell cycle and apoptosis analysis

C4-2B-299 and 22Rv-1-299 sublines and transfected PC-3 cells expressing miR-299 or scrambled RNA were induced with doxycycline and used for Propidium iodide staining. Cells were harvested at 48hr (PC-3) and 72hr (C4-2B-299 and 22Rv-1-299) post induction and fixed with cold 100% methanol. Cells were treated with 100µg/mL RNAse in 2% Bovine Serum Albumin (BSA) containing PBS. Cell were incubated at room temperature for 15 min and stained with 250µg/mL Propidium iodide in 2% BSA for 1hr at room temperature. Fluorescent cells were detected in CytoFlex S (Beckman Coulter) Flow Cytometer and analyzed using FlowJo software. For apoptosis assays, cells were harvested using 1X Trypsin EDTA-free (Fisher Scientific) 24h post induction and stained with 7-AAD and annexin V antibodies. Stained cells were analyzed in a CytoFlex S (Beckman Coulter) Flow Cytometer. Percentage of apoptotic cells were quantified using FlowJo software.

Scratch assay

Cells were induced with doxycycline and scratches were made with a pipet tip 24hr post induction. Cells were rinsed with PBS and incubated in media containing 5% FBS at 37°C. Migration of cells within the scratch was observed at the specified time 0 and 14 hrs for PC-3 cells and at 0 and 24hrs for C4-2B-299

and 22Rv-1-299 cells. Images were taken at 5X magnification using a Leica DMi8 inverted microscope and Leica LAS X software. ImageJ software was used to quantify the average width of each scratch. Analysis of cell migration was determined by subtracting the width of the scratch at the 14 and 24hrs from the width of the scratch at 0hr followed by analysis of the ratio of distance migrated by each cell line samples.

Western blot analysis

Whole cell lysates from induced and uninduced C4-2B-299 and 22Rv-1-299 cells and PC-3 cells expressing miR-299 or scrambled RNA were prepared at 48hrs and 72hrs post induction with doxycycline. Similarly, PC-3 cells expressing miR-299 or scrambled RNA at 24 hours post induction with doxycycline were treated with 25nM DTX. After 48 hours of treatment whole cell lysates were prepared. Fifty μg of extracted proteins was separated in a 10% Bis-Tris gel and transferred to activated PVDF membranes. Immunoblotting was performed with primary antibodies specific for AR (LSBio), VEGFA (Santa Cruz Biotechnology) and GAPDH (Sigma Aldrich). Antibodies for α-tubulin, Slug, Vimentin, E-Cadherin, Cyclin D1, Cyclin B1, pan-Akt, phospho-Akt, PRAS40 and phospho-PRAS40 were obtained from Cell Signaling.

Immunohistochemistry

Briefly, 5μM sections of tumor tissues were deparaffinized and rehydrated using xylene and ethanol. Next, antigen retrieval was performed by boiling the slides immersed in 1X TE buffer for 13 minutes followed by cooling the slides for 20 minutes. Then Ki67 rabbit monoclonal antibodies conjugated with Alexa (R) 488 (Cell Signaling) and mouse monoclonal cytokeratin 8 antibodies (Santa Cruz Biotechnology) were used as primary antibodies. Anti-mouse antibodies conjugated with Texas Red Dye (Vector Laboratories) was used as the secondary antibodies and slides were mounted using DAPI Fluoromount-G (SouthernBiotech). All slides were imaged using the Leica SP5 confocal microscope Percentage of Ki67 positive cells were then quantified using ImageJ software.

Figure Legend:

Figure S1. Expression of miR-299-3p in C4-2B, 22Rv-1 and PC-3 cells. A&B. Bright-field images showing exclusive expression of miR-299-3p in C4-2B and 22Rv-1 stable cells upon doxycycline treatment (**A**) and expression of miR-299-3p or the scr RNA in PC-3 cells (**B**). **C&D.** Average fold-change in expression of miR-299-3p in stably transfected C4-2B and 22Rv-1 cells (**C**) and transiently transfected PC-3 cells (**D**). Data show the mean±SD of more than three independent assays.

Figure S2. Expression of miR-299-3p induced G1/S arrest and reduced expression of cyclin D. A. Two parameter histogram of cell cycle of induced 22Rv-1 cells overexpressing miR-299-3p compared to uninduced cells. Cell cycle analysis performed after 72h of induction showing a G1/S arrest. **B.** Comparative analysis of percentage of cells showing a significant increase in number of cells in G1 and a significant decrease in number of cells in S-phase. Data show the mean±SD of at least 3 independent assays. **C**. Representative images of immunoblot analysis of CCND1 (G1 marker) and α-tubulin as the internal control. **D**. Comparative analysis of CCND1 and expression showing significant reduction in expression of these markers in induced 22Rv-1 cells expressing miR-299-3p at 72hr post induction. Values were normalized to the internal control. Data represent mean±SD of three independent experiments.

Figure S3. Expression of miR-299-3p improved drug sensitivity in AR-positive and -negative PCa cells. A&B. 22Rv-1-299-3p cells were induced or left uninduced and treated with ENZ and DTX and sensitivity to drugs was measured through MTS assays. DMSO was used as the vehicle control. **A**. Percent increase in cell death in induced 22Rv-1-299-3p cells treated with 20μM and 40 μM ENZ compared to uninduced cells and DMSO. Data represent mean±SD of at least 3 independent experiments in triplicates. **B.** Percent increase in cell death in induced 22Rv-1 cells expressing miR-299-3p treated with 2nM and 20nM DTX compared to uninduced cells and DMSO. Data represent mean±SD of at least

3 independent experiments in triplicates. **C.** PC-3 cells transfected with miR-299-3p or Scramble RNA were induced and treated with DTX followed by measurement of drug sensitivity through MTS assays. DMSO was used as the vehicle control. Percent increase in cell death in induced PC-3 cells expressing miR-299-3p treated with 10nM and 25nM DTX compared to induced control and DMSO. Data represent mean±SD of at least 3 independent experiments in triplicates. **D.** LNCaP-104S cells transfected with miR-299-3p or Scramble RNA were induced and treated with ENZ followed by measurement of drug sensitivity through MTS assays. DMSO was used as the vehicle control. Percent increase in cell death in induced LNCaP-104S cells expressing miR-299-3p treated with 20uM and 40uM ENZ compared to induced control and DMSO. Data represent mean±SD of at least 3 independent experiments in triplicates.

Figure S4. Reduced expression of Ki67 in miR-299-3p expressing tumors. Immunofluorescence analysis of the expression of Ki67 in mice tumor tissues from uninduced and induced groups. Representative images show a noticeable reduction in $Ki67^{+}$ cells in induced tumors compared to the uninduced tumor tissues. No change in expression of cytokeratin 8 could be noted. DAPI was used as the nuclear stain. H&E stained images show the histology of tumor tissues. Scale bar: 10μm

Figure S5. A&B. Quantitative real-time PCR showing reduced expression of AR and PSA in induced C4-2B-299-3p and 22Rv-1-299-3p cells compared to uninduced cells. Data show mean±SD of three separate experiments. **C**. Western blot analysis showing reduced expression of VEGFA in lysates of induced 22Rv-1-299-3p compared to uninduced cells. **D**. Densitometric analysis of VEGFA expression after normalization with the internal control. Alpha tubulin was used as the internal control. Data show mean±SD of three separate experiments. **E**. Quantitative RT-PCR analysis showing reduced VEGFA mRNA expression in induced 22Rv-299 cells compared to uninduced controls and in transiently transfected miR-299-3p expressing PC-3 cells compared to Scramble RNA. Data show mean±SD of three separate experiments. **F.** Western blot representative image showing increased AR and VEGFA in lysates of miR-299-3p inhibitor transfected induced C4-2B-299-3p cells compared to control inhibitor

transfected cells. GAPDH was used as the internal control. **G.** Densitometric analysis of the AR and VEGFA concentrations normalized to the internal control. Data represent mean±SD of at least three independent assays. **H.** Western Blot representative image showing increased expression of full length AR and AR-v-7 in lysates of miR-299-3p inhibitor transfected induced 22Rv-299-3p cells compared to control inhibitor transfected cells. GAPDH was used as the internal control. **I.** Densitometric analysis of full-length AR and AR-v-7 concentrations normalized to the internal control. Data represent mean±SD of at least three independent assays.

Figure S6. Expression of miR-299-3p decreased migration and promoted an epithelial phenotype. Migration and expression of EMT markers were compared between induced and uninduced 22Rv-1 cells stably expressing miR-299-3p. **A.** Bright field images taken at 0 hours and 24 hours after making the scratch showing the relative rates of migration of 22Rv-1-299-3p induced stable line compared to the uninduced control. Scale bar: 200μm. **B**. Relative rates of cell migration. Data presented as the ratio of the distance traversed by the uninduced or induced scr RNA control cells compared to induced cells expressing miR-299-3p. Data show the mean±SD of minimum three independent assays. **C.** Representative images of the immunoblot analysis of Slug and E-cadherin in induced and uninduced 22Rv-1-299-3p cells. Antibodies against α-tubulin was used as the internal control. **D.** Densitometric analysis showing reduced expression of Slug and increased induced scrRNA control cells. Values were normalized to the internal control. Data represent mean±SD of at least 3 independent assays. **E.** Quantitative RT-PCR showing decreased TGFB3 mRNA expression in induced 22Rv-1-299-3p cells expressing miR-299-3p compared to uninduced cells. Data show the mean±SD of three independent assays.

Table 1: Patient criteria

Patient #	Age	PSA pre-surgery	Gleason Score	Clinical Stage
1	49YR	10.9	3+4=7	PT3aNOMX
2	65YR	6.3	3+4=7	PT2cNXMX
3	65YR	5.5	3+4=7	PT3aN0MX
4	70YR	6.3	3+4=7	PT2cNxMx
5	61YR	12.7	5+4=9	PT2cN0MX
6	67YR	6.2	3+4=7	PT2CR1NXMX
7	68YR	9.03	4+4=8	PT3aN0MX
8	71YR	6.4	4+5=9	PT3aNOMX
9	52YR	9	4+3=7	PT3aR1NxMx
10	61YR	6.4	3+4=7	PT2aNXMX
11	61YR	6.4	3+4=7	PT2aNXMX
12	65YR	12	3+4=7	PT2xN0MX
13	69YR	6.42	3+3=6	pT2cNXMX
14	54YR	87.4	3+3=6	PT3aN0MX
15	56YR	5.2	3+4=7	PT2cNXMX
16	53YR	4.3	3+3=6	T2cR1NXMX
17	53YR	12.2	3+4=7	PT3N0MX
18	48YR	6.5	3+4=7	T1cNXMX

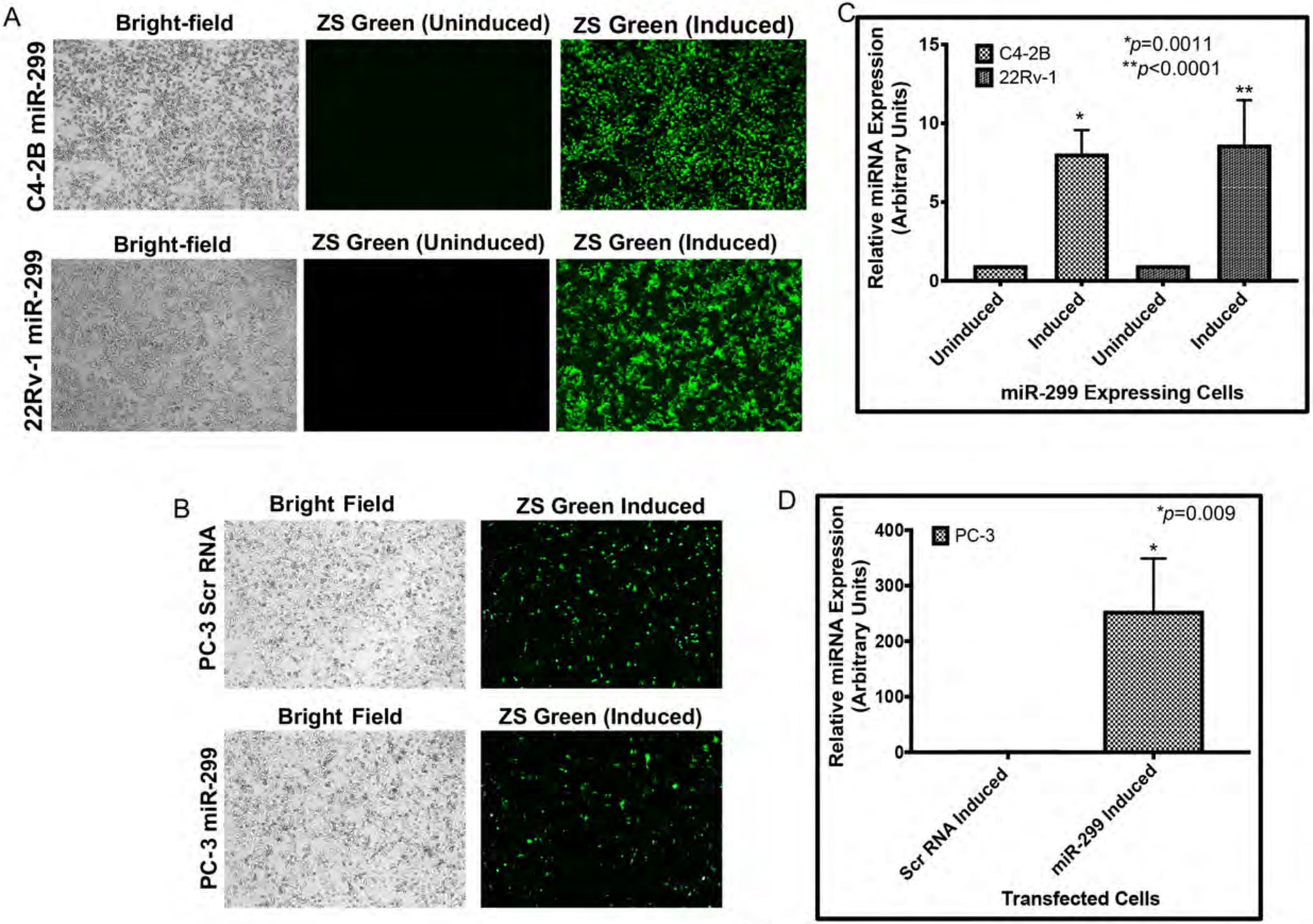
A
Bright-field
ZS Green (Uninduced)
ZS Green (Induced)
C4-2B miR-299
22Rv-1 miR-299
B
Bright Field
ZS Green Induced
ZS Green (Induced)
PC-3 Scr RNA
PC-3 miR-299
C
C4-2B
22Rv-1
*p=0.0011
**p<0.0001
Relative miRNA Expression (Arbitrary Units)
15
10
5
0
Uninduced
Induced
Uninduced
Induced
miR-299 Expressing Cells
D
PC-3
*p=0.009
Relative miRNA Expression (Arbitrary Units)
400
300
200
100
0
Scr RNA Induced
miR-299 Induced
Transfected Cells

Fig. S1

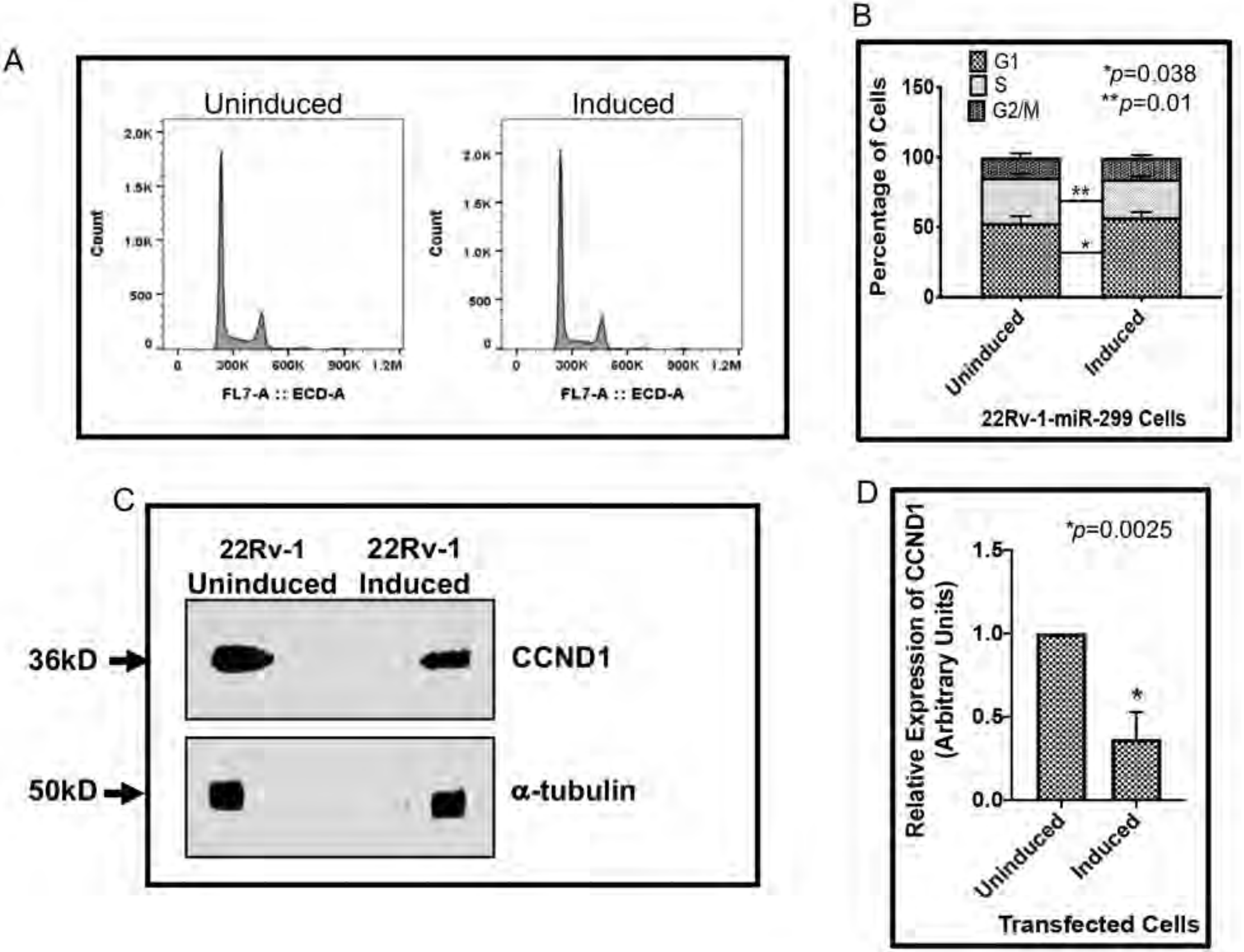
A
Uninduced
Induced
Count
FL7-A :: ECD-A
B
G1
S
G2/M
*p=0.038
**p=0.01
Percentage of Cells
Uninduced
Induced
22Rv-1-miR-299 Cells
C
22Rv-1 Uninduced
22Rv-1 Induced
36kD
CCND1
50kD
α-tubulin
D
*p=0.0025
Relative Expression of CCND1 (Arbitrary Units)
Uninduced
Induced
Transfected Cells

Fig. S2

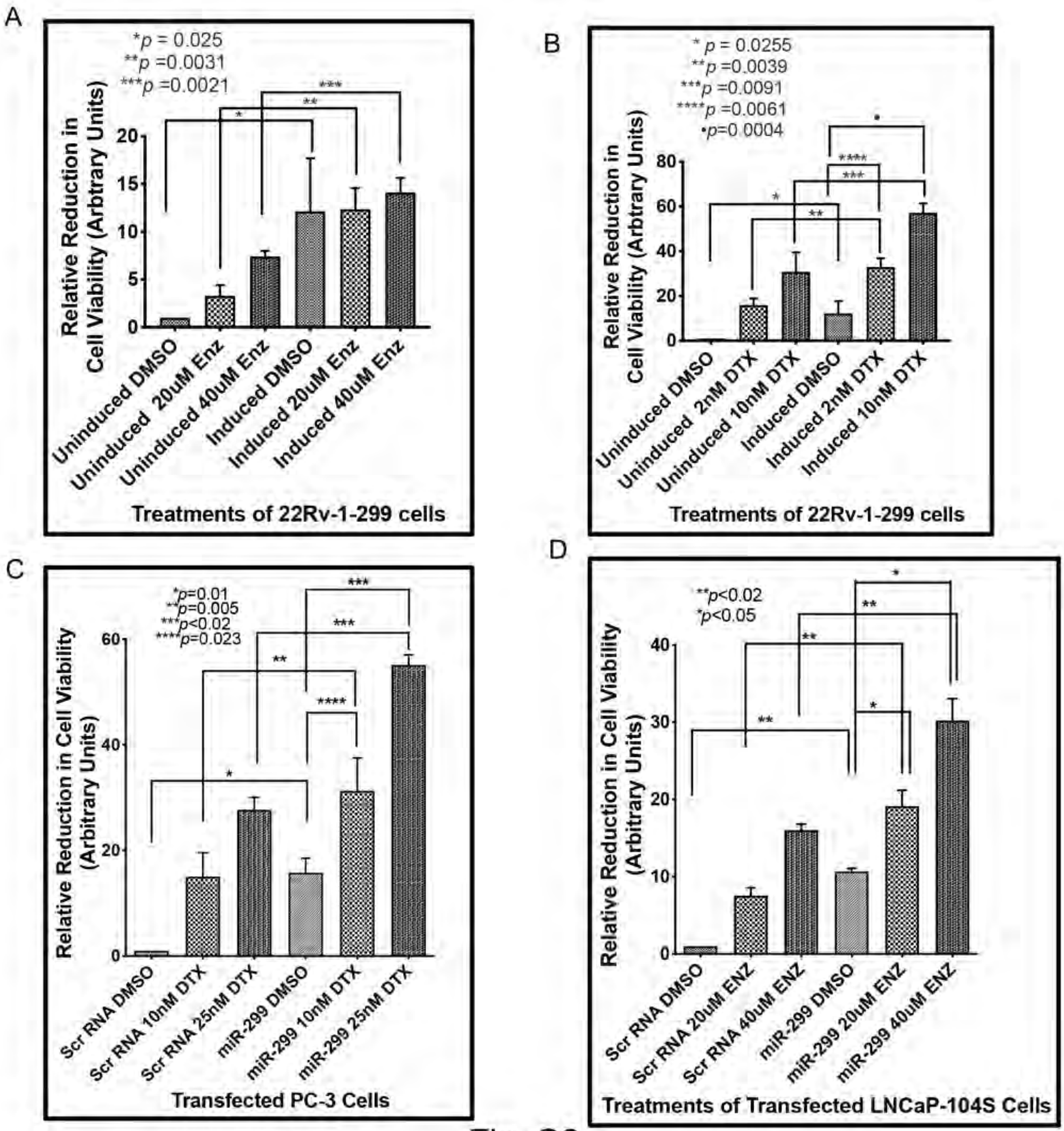

A
*p = 0.025
**p =0.0031
***p =0.0021
Relative Reduction in Cell Viability (Arbtrary Units)
Uninduced DMSO
Uninduced 20uM Enz
Uninduced 40uM Enz
Induced DMSO
Induced 20uM Enz
Induced 40uM Enz
Treatments of 22Rv-1-299 cells
B
* p = 0.0255
**p =0.0039
***p =0.0091
****p =0.0061
•p=0.0004
Relative Reduction in Cell Viability (Arbtrary Units)
Uninduced DMSO
Uninduced 2nM DTX
Uninduced 10nM DTX
Induced DMSO
Induced 2nM DTX
Induced 10nM DTX
Treatments of 22Rv-1-299 cells
C
*p=0.01
**p=0.005
***p<0.02
****p=0.023
Relative Reduction in Cell Viability (Arbitrary Units)
Scr RNA DMSO
Scr RNA 10nM DTX
Scr RNA 25nM DTX
miR-299 DMSO
miR-299 10nM DTX
miR-299 25nM DTX
Transfected PC-3 Cells
D
**p<0.02
*p<0.05
Relative Reduction in Cell Viability (Arbitrary Units)
Scr RNA DMSO
Scr RNA 20uM ENZ
Scr RNA 40uM ENZ
miR-299 DMSO
miR-299 20uM ENZ
miR-299 40uM ENZ
Treatments of Transfected LNCaP-104S Cells

Fig. S3

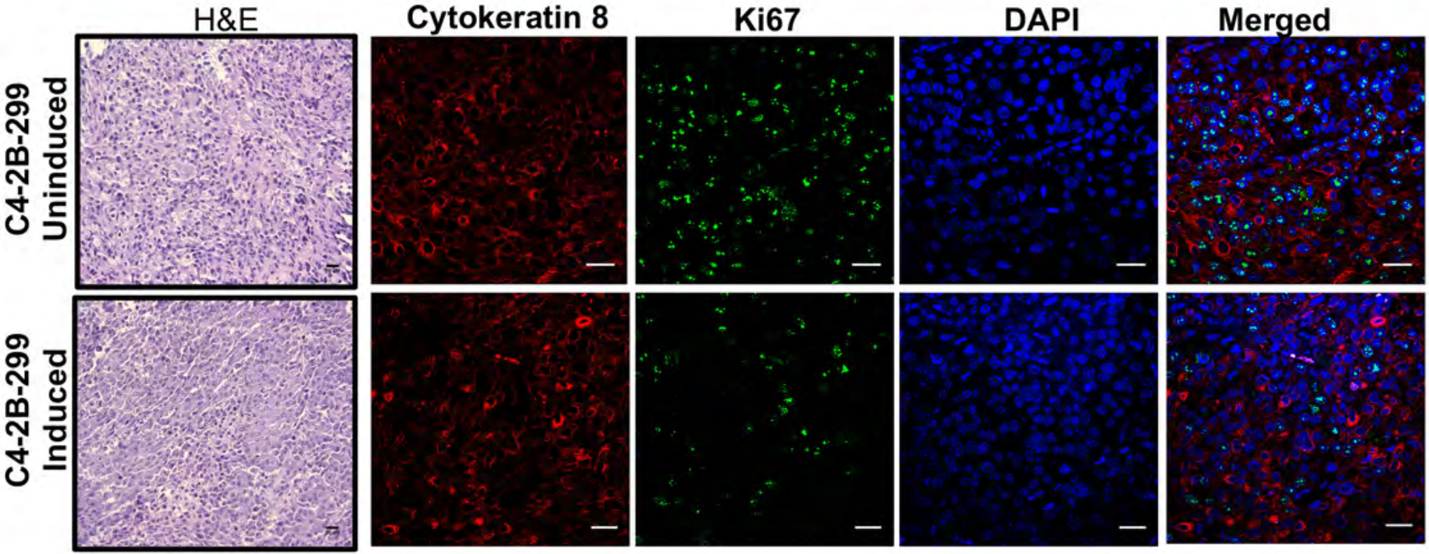
H&E
Cytokeratin 8
Ki67
DAPI
Merged
C4-2B-299 Uninduced
C4-2B-299 Induced

Fig. S4

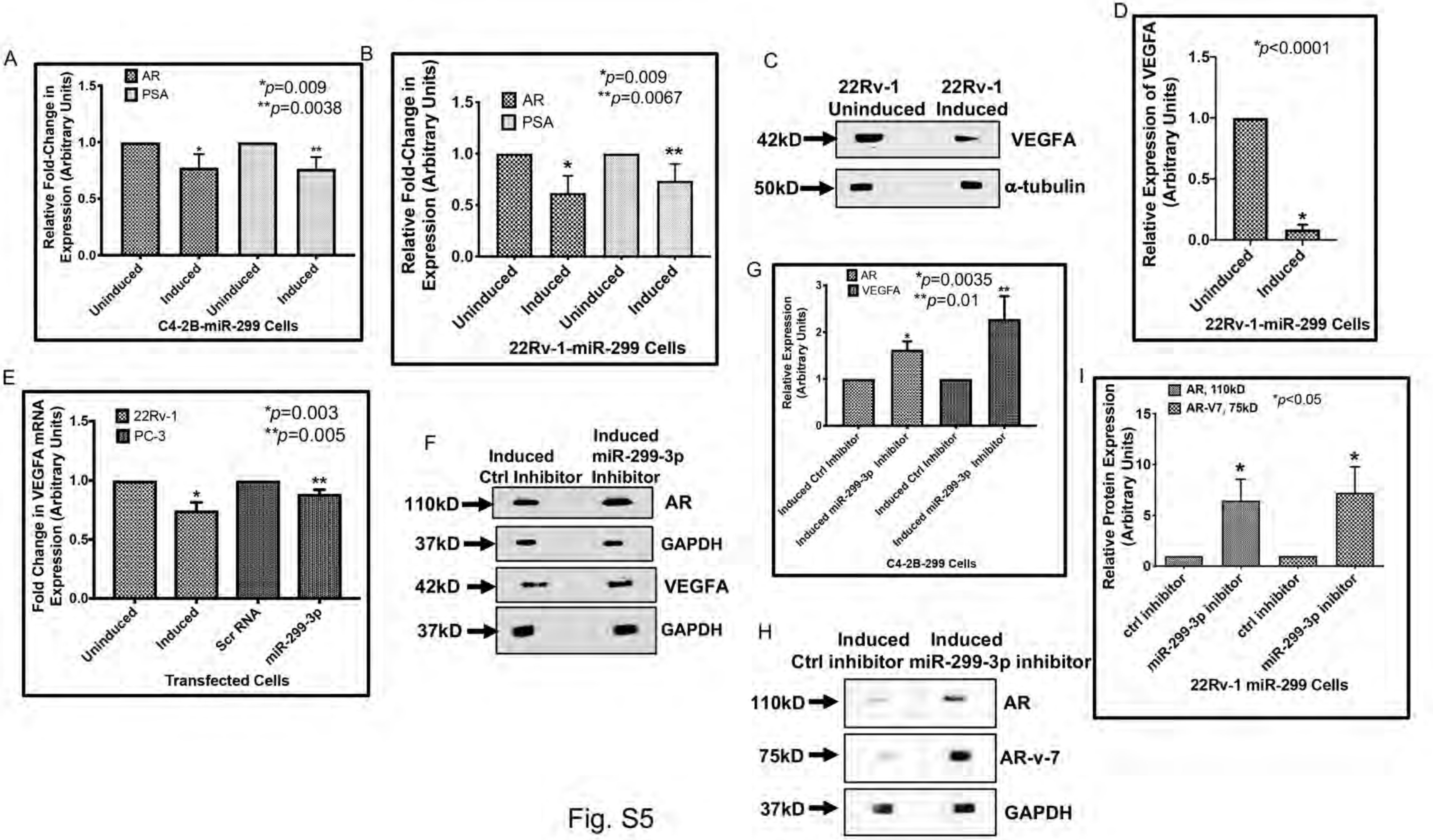
A
AR
PSA
*p=0.009
**p=0.0038
Relative Fold-Change in Expression (Arbitrary Units)
1.5
1.0
0.5
0.0
Uninduced
Induced
Uninduced
Induced
C4-2B-miR-299 Cells
B
AR
PSA
*p=0.009
**p=0.0067
Relative Fold-Change in Expression (Arbitrary Units)
Uninduced
Induced
Uninduced
Induced
22Rv-1-miR-299 Cells
C
22Rv-1 Uninduced
22Rv-1 Induced
42kD
VEGFA
50kD
α-tubulin
D
*p<0.0001
Relative Expression of VEGFA (Arbitrary Units)
Uninduced
Induced
22Rv-1-miR-299 Cells
E
22Rv-1
PC-3
*p=0.003
**p=0.005
Fold Change in VEGFA mRNA Expression (Arbitrary Units)
Uninduced
Induced
Scr RNA
miR-299-3p
Transfected Cells
F
Induced Ctrl Inhibitor
Induced miR-299-3p Inhibitor
110kD
AR
37kD
GAPDH
42kD
VEGFA
37kD
GAPDH
G
AR
VEGFA
*p=0.0035
**p=0.01
Relative Expression (Arbitrary Units)
Induced Ctrl Inhibitor
Induced miR-299-3p Inhibitor
Induced Ctrl Inhibitor
Induced miR-299-3p Inhibitor
C4-2B-299 Cells
H
Induced Ctrl inhibitor
Induced miR-299-3p inhibitor
110kD
AR
75kD
AR-v-7
37kD
GAPDH
I
AR, 110kD
AR-V7, 75kD
*p<0.05
Relative Protein Expression (Arbitrary Units)
15
10
5
0
ctrl inhibitor
miR-299-3p inibitor
ctrl inhibitor
miR-299-3p inibitor
22Rv-1 miR-299 Cells

Fig. S5

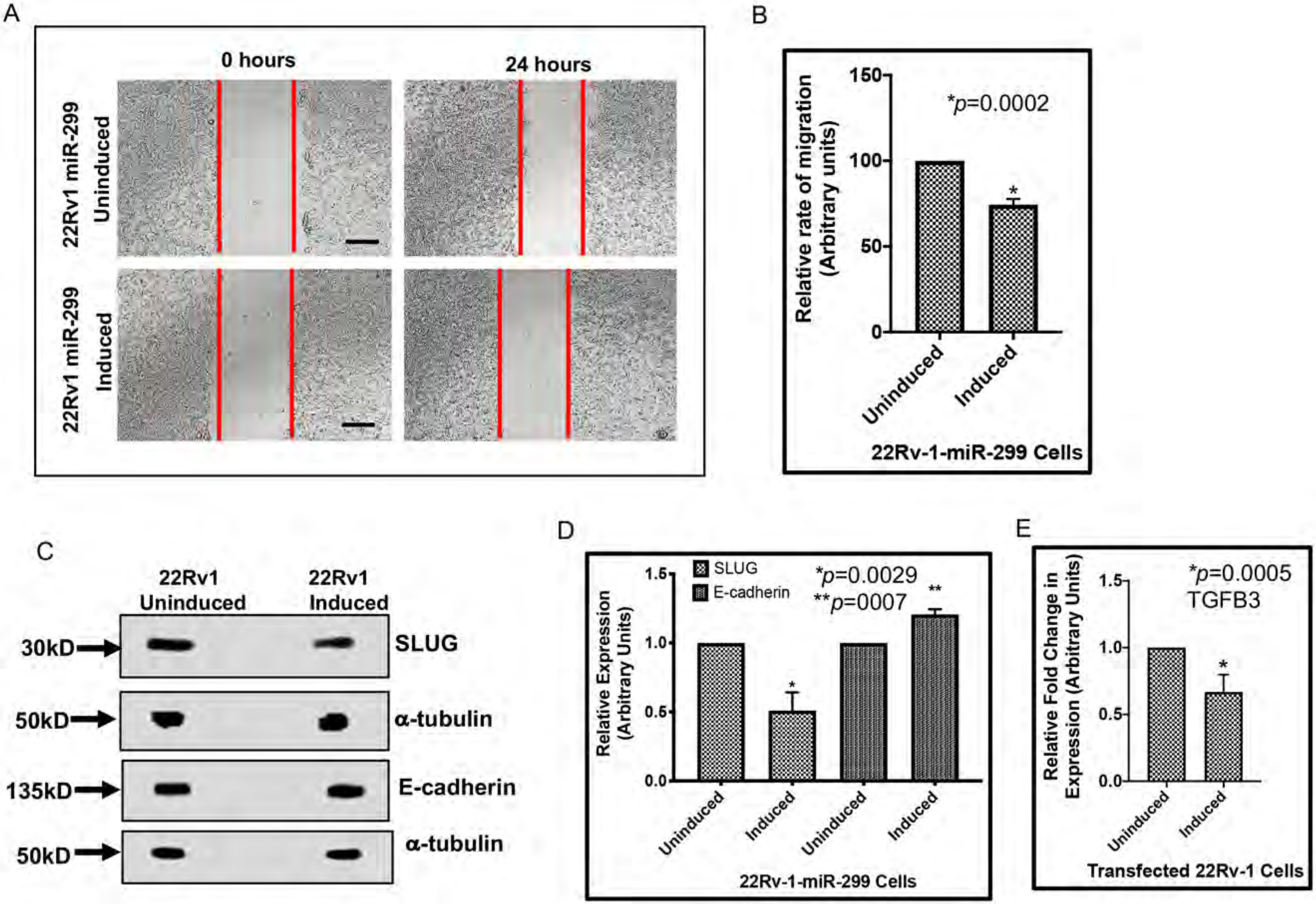
A
0 hours
24 hours
22Rv1 miR-299 Uninduced
22Rv1 miR-299 Induced
B
Relative rate of migration (Arbitrary units)
*p=0.0002
150
100
50
0
Uninduced
Induced
22Rv-1-miR-299 Cells
C
22Rv1 Uninduced
22Rv1 Induced
30kD
SLUG
50kD
α-tubulin
135kD
E-cadherin
50kD
α-tubulin
D
SLUG
E-cadherin
*p=0.0029
**p=0007
Relative Expression (Arbitrary Units)
1.5
1.0
0.5
0.0
Uninduced
Induced
Uninduced
Induced
22Rv-1-miR-299 Cells
E
*p=0.0005
TGFB3
Relative Fold Change in Expression (Arbitrary Units)
1.5
1.0
0.5
0.0
Uninduced
Induced
Transfected 22Rv-1 Cells

Fig. S6

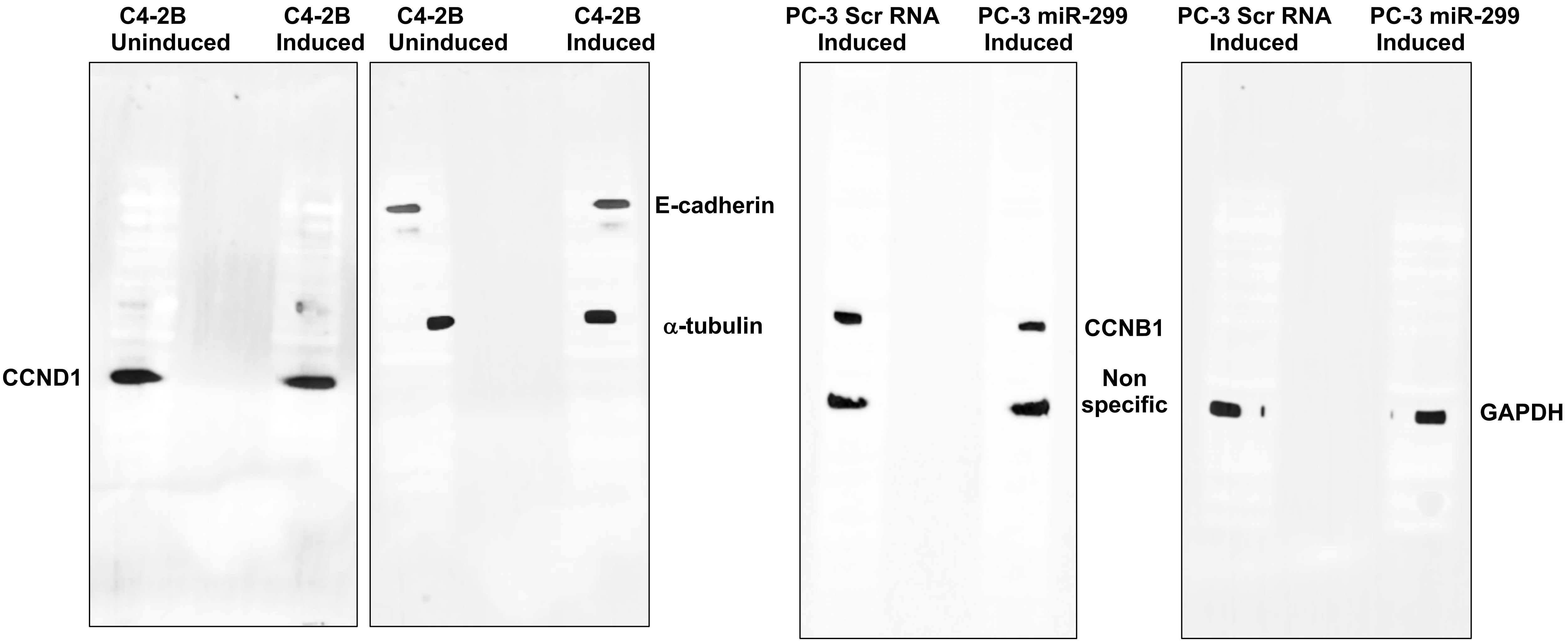

The blot was re-probed for E-cadherin

Fig. 2C

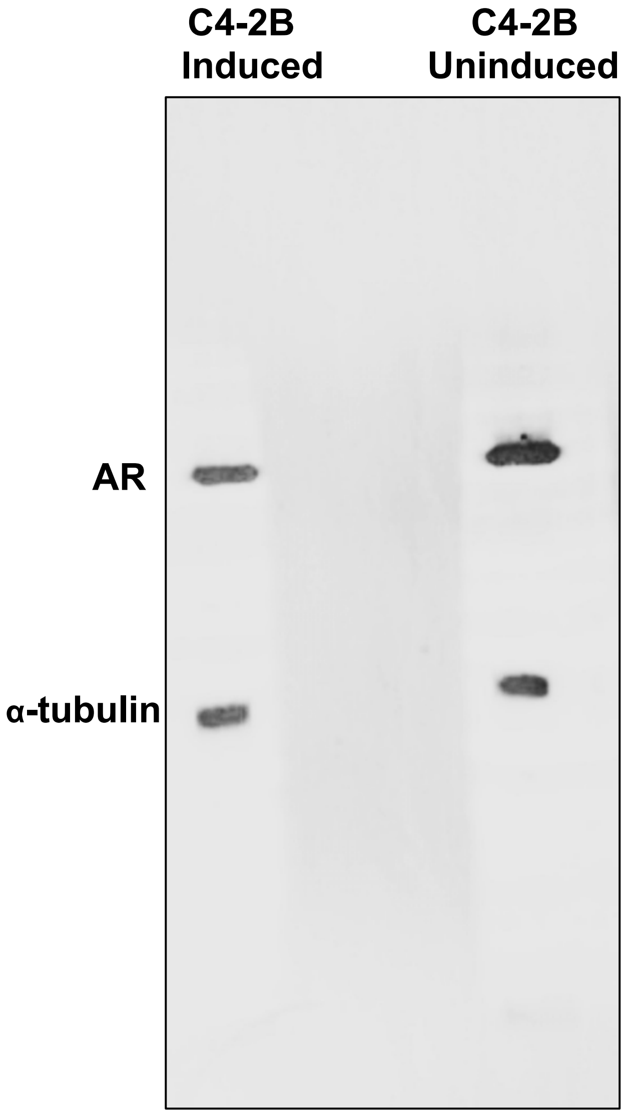
C4-2B
Induced
C4-2B
Uninduced
AR
α-tubulin

Fig. 4A

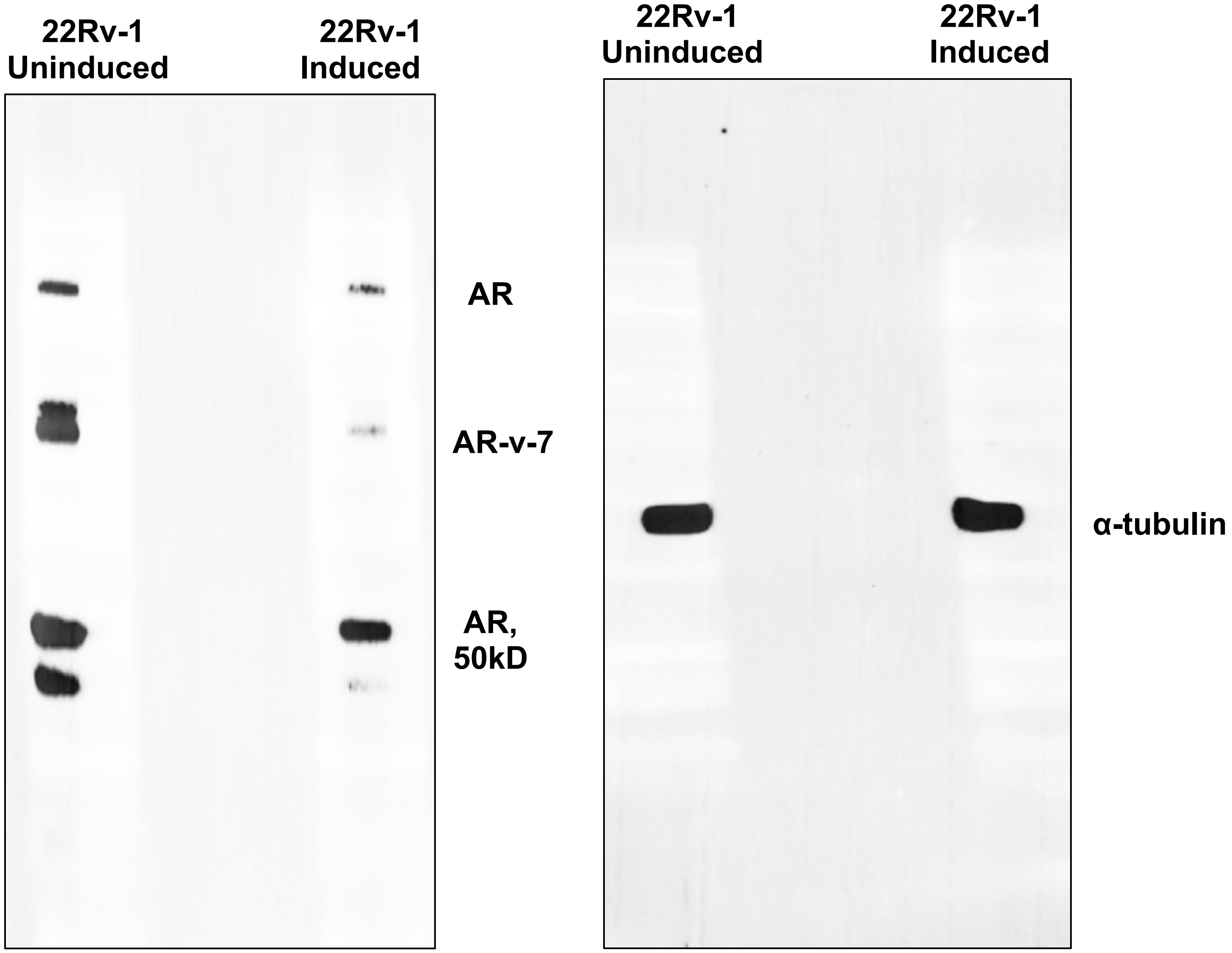
22Rv-1
Uninduced
22Rv-1
Induced
AR
AR-v-7
AR,
50kD
22Rv-1
Uninduced
22Rv-1
Induced
α-tubulin

Fig. 4A

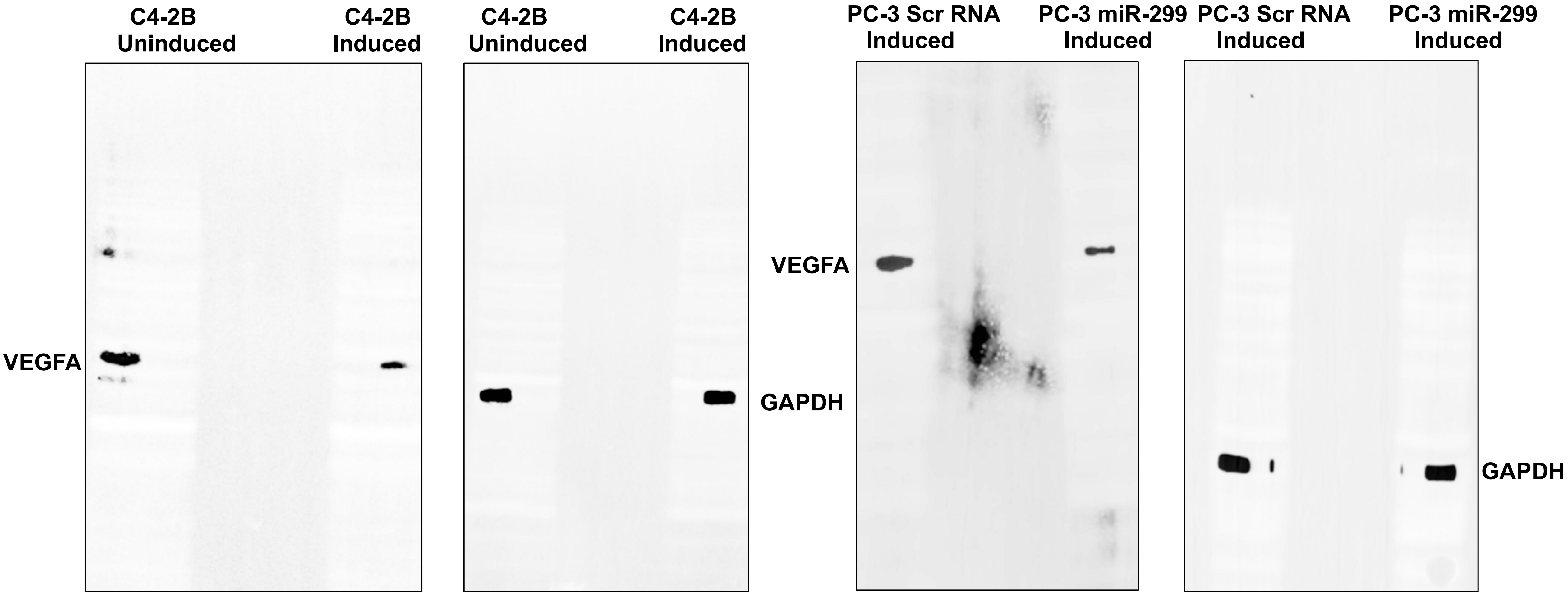
C4-2B Uninduced
C4-2B Induced
C4-2B Uninduced
C4-2B Induced
PC-3 Scr RNA Induced
PC-3 miR-299 Induced
PC-3 Scr RNA Induced
PC-3 miR-299 Induced
VEGFA
GAPDH
VEGFA
GAPDH

Fig. 4C

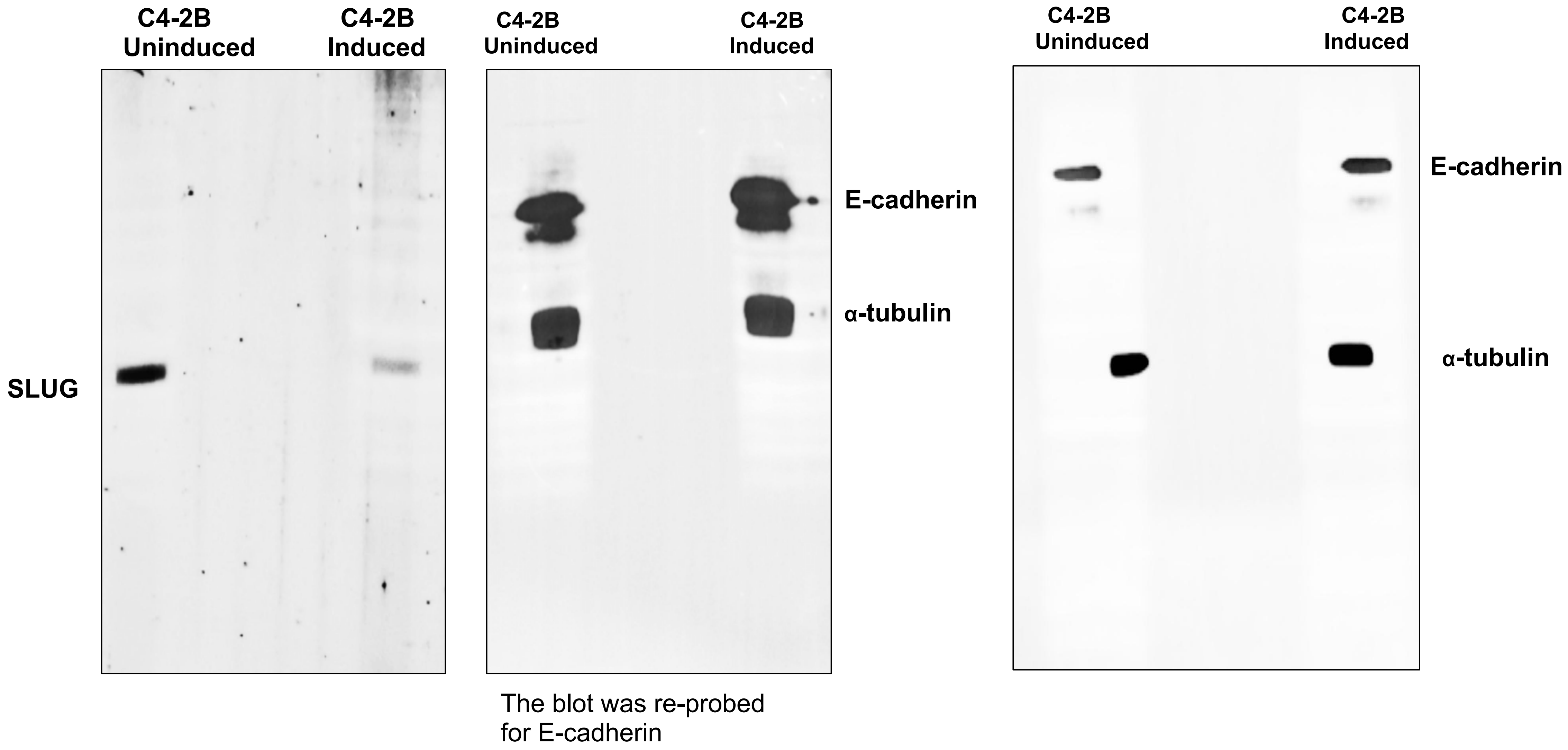
C4-2B Uninduced
C4-2B Induced
SLUG
C4-2B Uninduced
C4-2B Induced
E-cadherin
α-tubulin
The blot was re-probed for E-cadherin
C4-2B Uninduced
C4-2B Induced
E-cadherin
α-tubulin

Fig. 5D

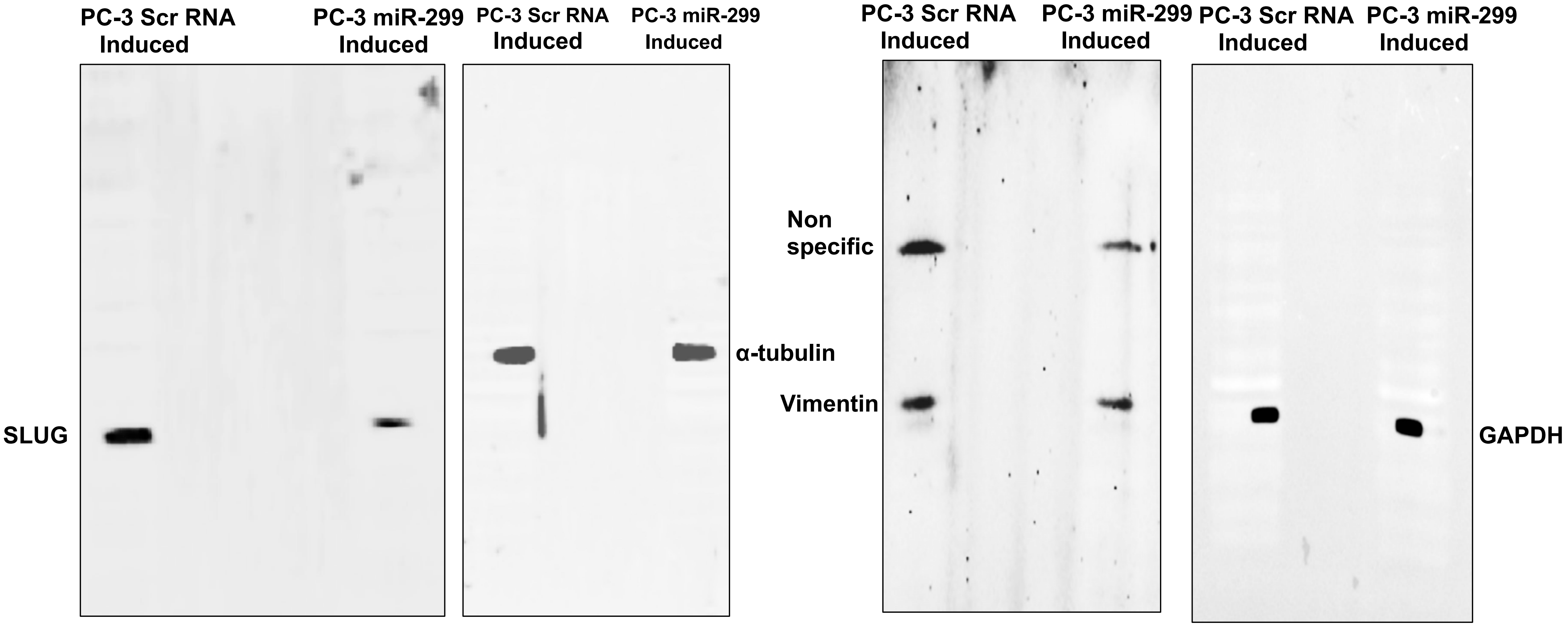
PC-3 Scr RNA Induced
PC-3 miR-299 Induced
PC-3 Scr RNA Induced
PC-3 miR-299 Induced
PC-3 Scr RNA Induced
PC-3 miR-299 Induced
PC-3 Scr RNA Induced
PC-3 miR-299 Induced
SLUG
α-tubulin
Non specific
Vimentin
GAPDH

Fig. 5E

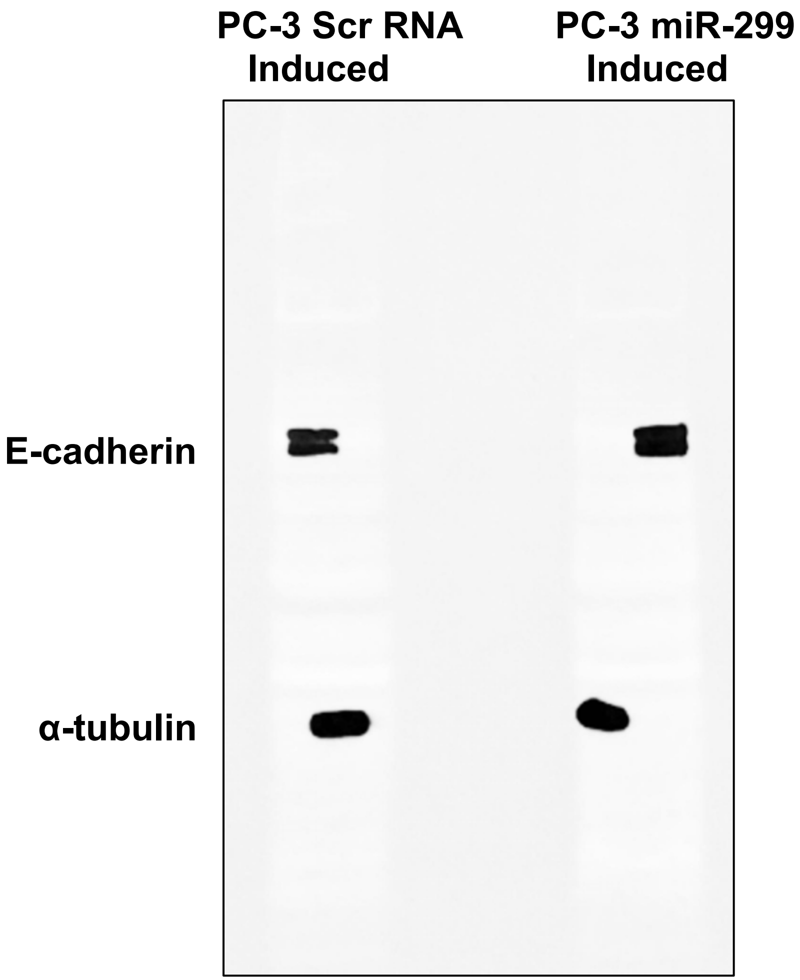
PC-3 Scr RNA Induced
PC-3 miR-299 Induced
E-cadherin
α-tubulin

Fig. 5E

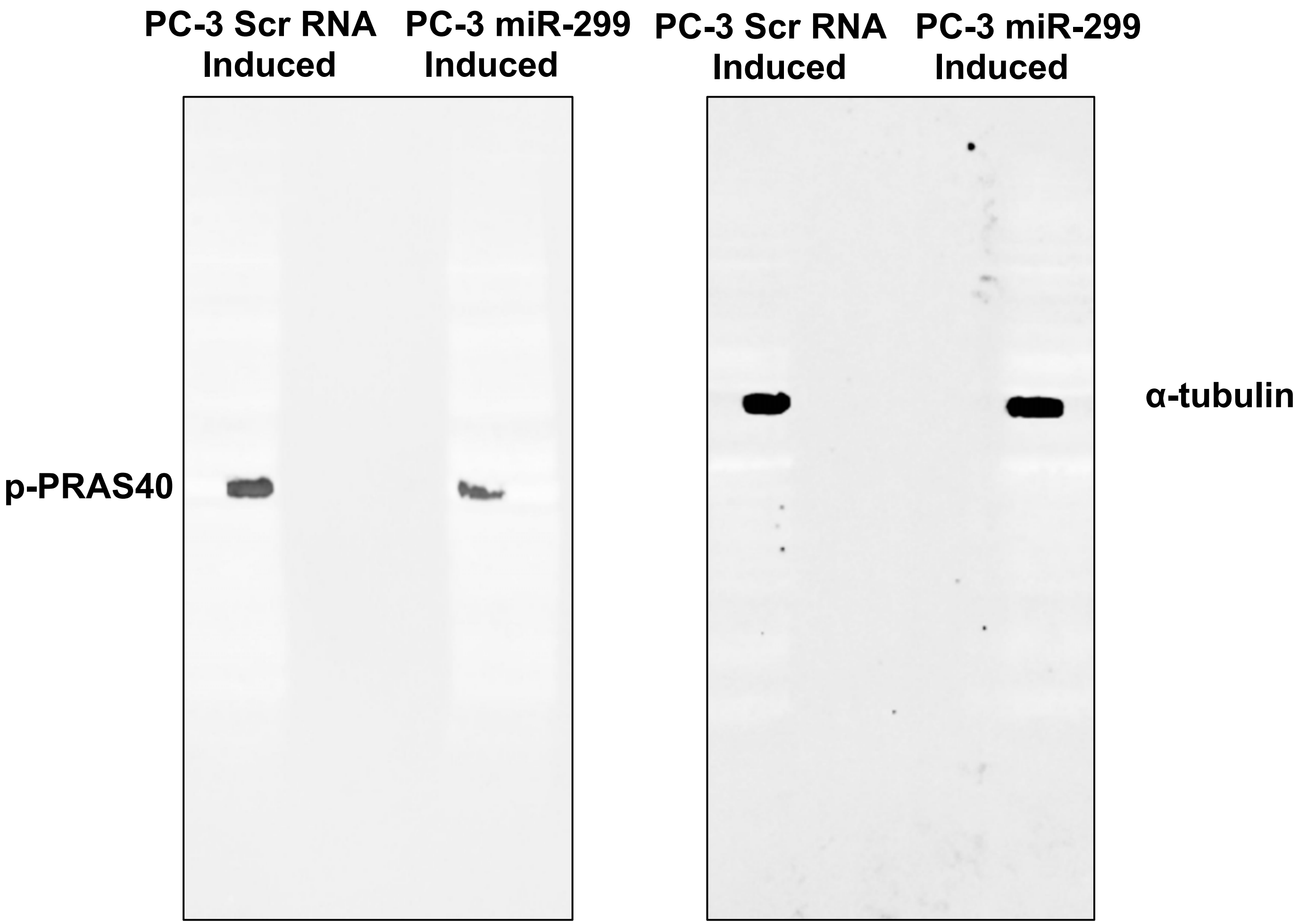
PC-3 Scr RNA Induced
PC-3 miR-299 Induced
p-PRAS40
PC-3 Scr RNA Induced
PC-3 miR-299 Induced
α-tubulin

Fig. 6A

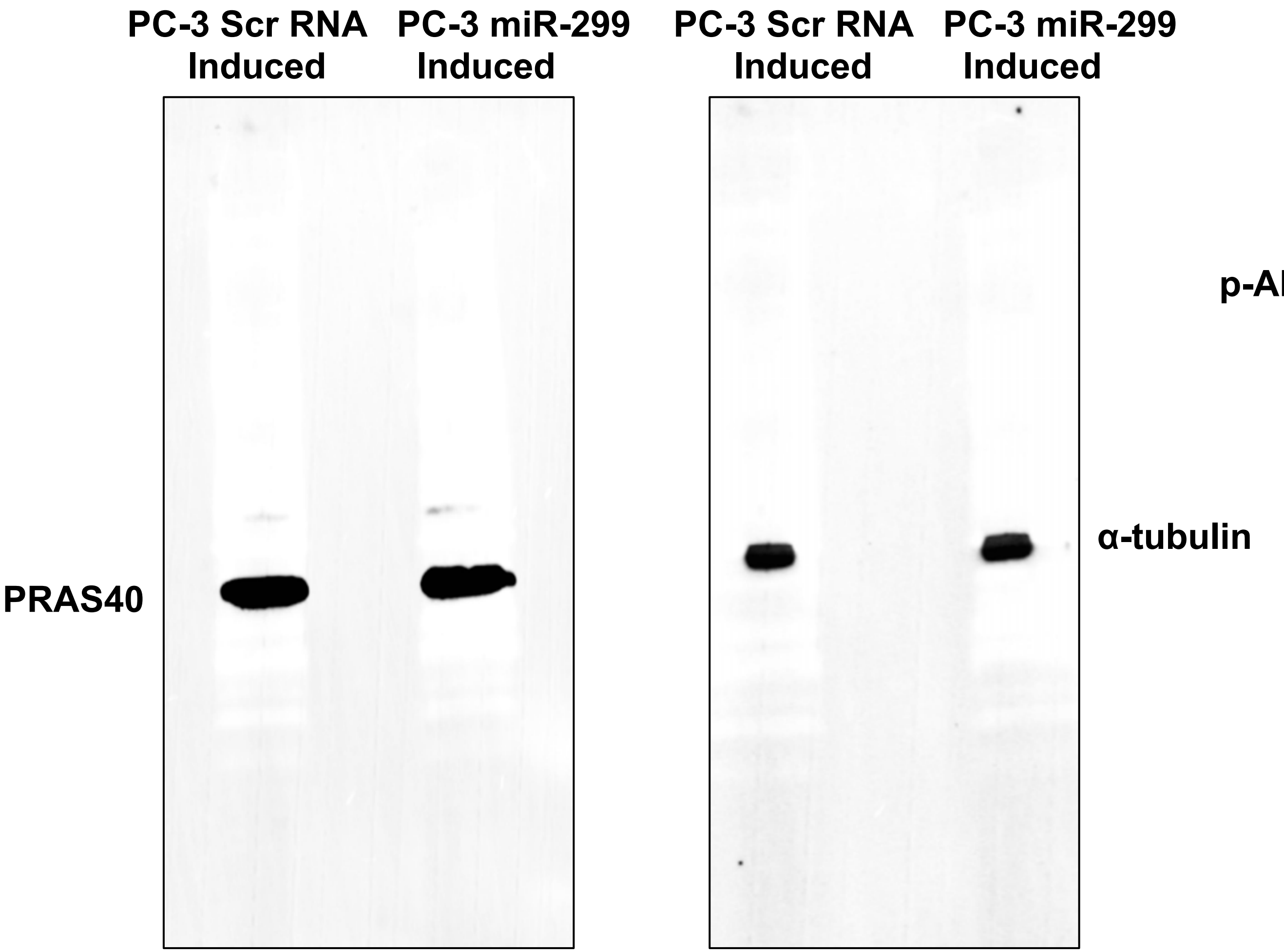
PC-3 Scr RNA Induced
PC-3 miR-299 Induced
PC-3 Scr RNA Induced
PC-3 miR-299 Induced
PRAS40
α-tubulin

Fig. 6A

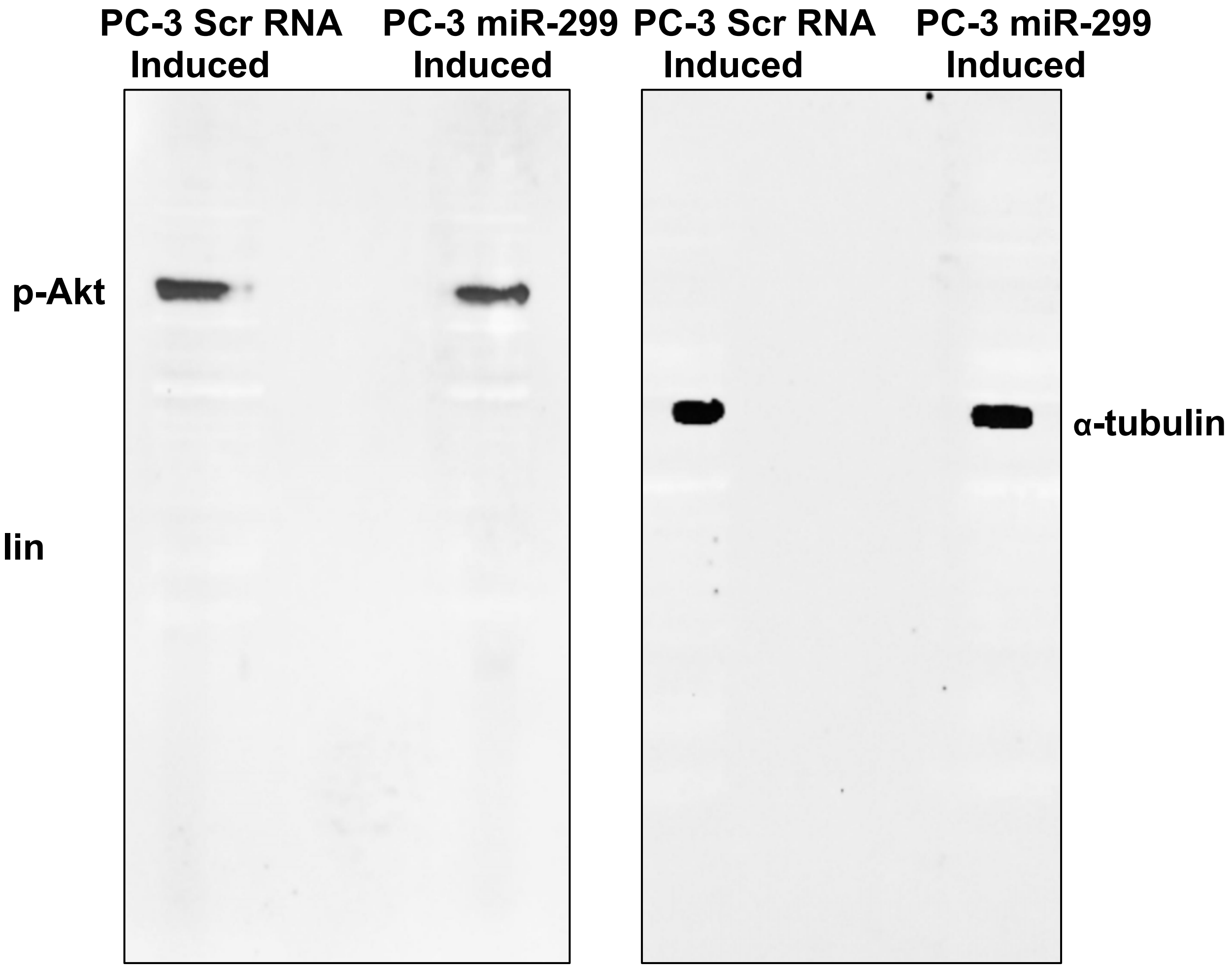
PC-3 Scr RNA Induced
PC-3 miR-299 Induced
PC-3 Scr RNA Induced
PC-3 miR-299 Induced
p-Akt
α-tubulin

Fig. 6B

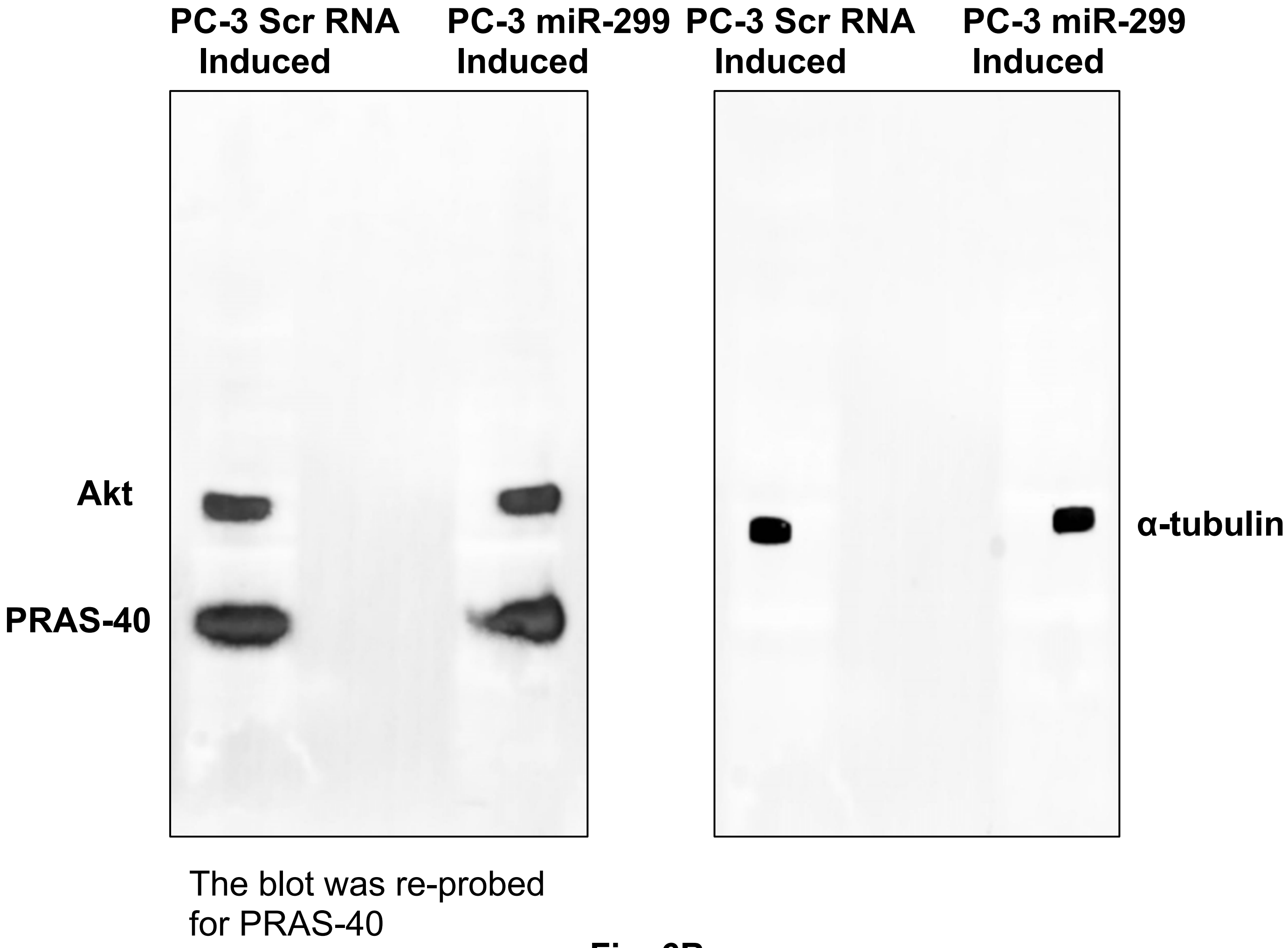

PC-3 Scr RNA Induced
PC-3 miR-299 Induced
PC-3 Scr RNA Induced
PC-3 miR-299 Induced
Akt
PRAS-40
α-tubulin
The blot was re-probed for PRAS-40

Fig. 6B

Supplementary Figures

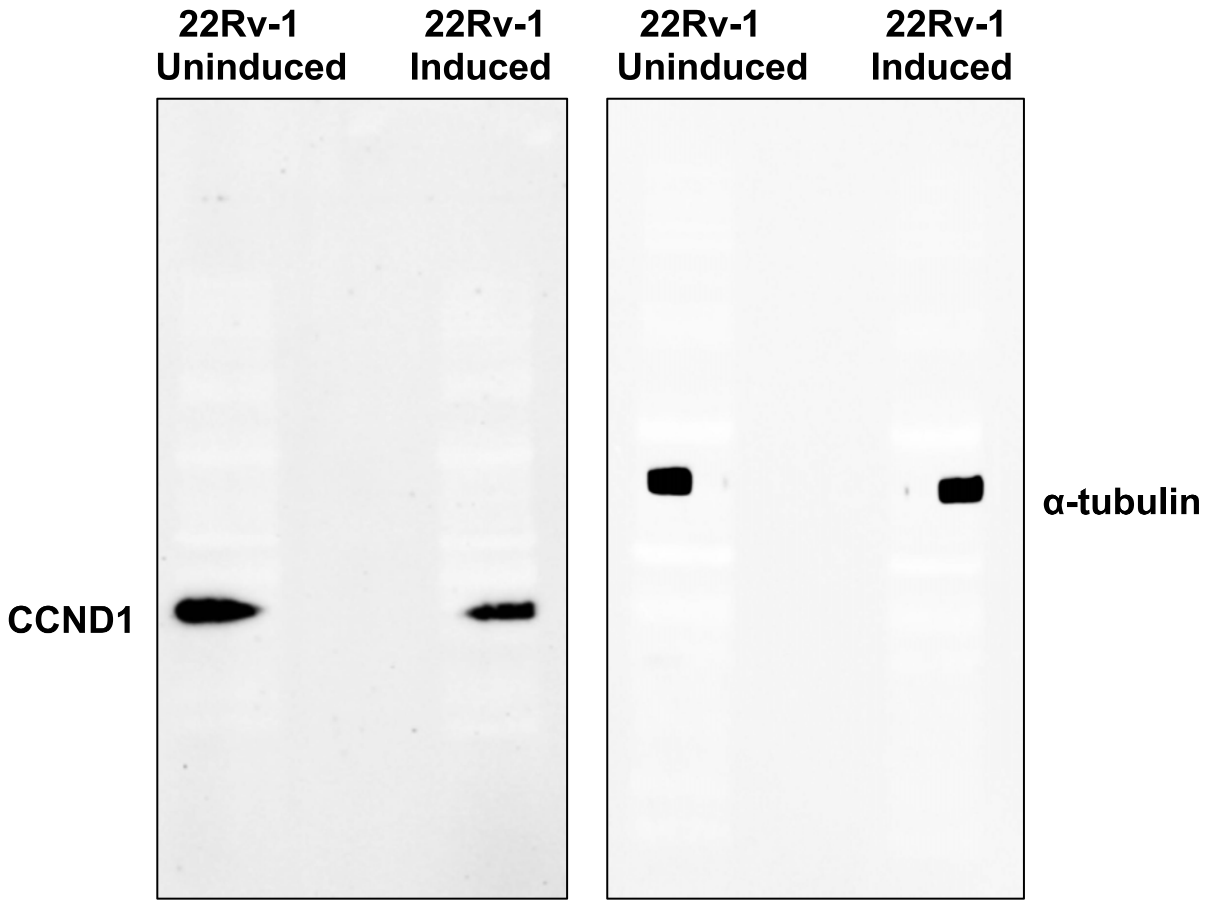

Fig. S2C

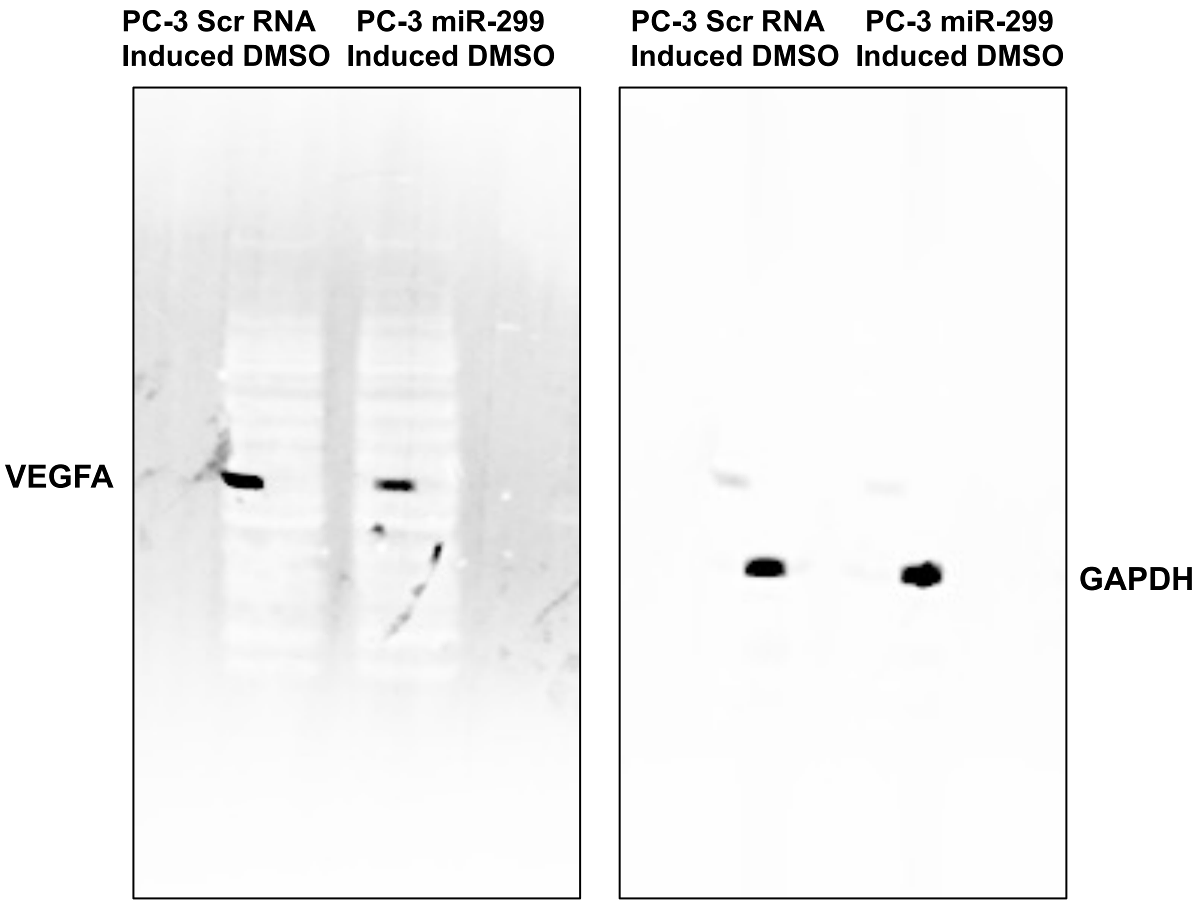

Fig. S3E

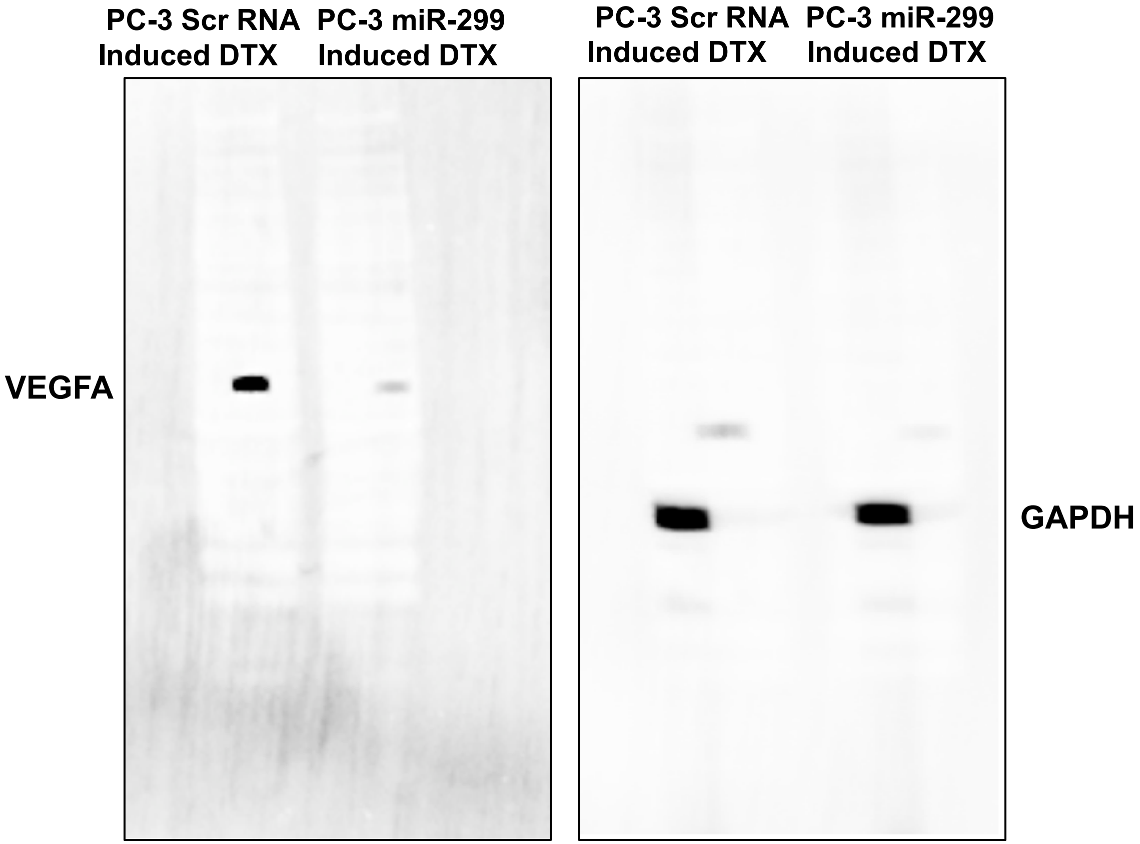
PC-3 Scr RNA Induced DTX
PC-3 miR-299 Induced DTX
PC-3 Scr RNA Induced DTX
PC-3 miR-299 Induced DTX
VEGFA
GAPDH

Fig. S3E

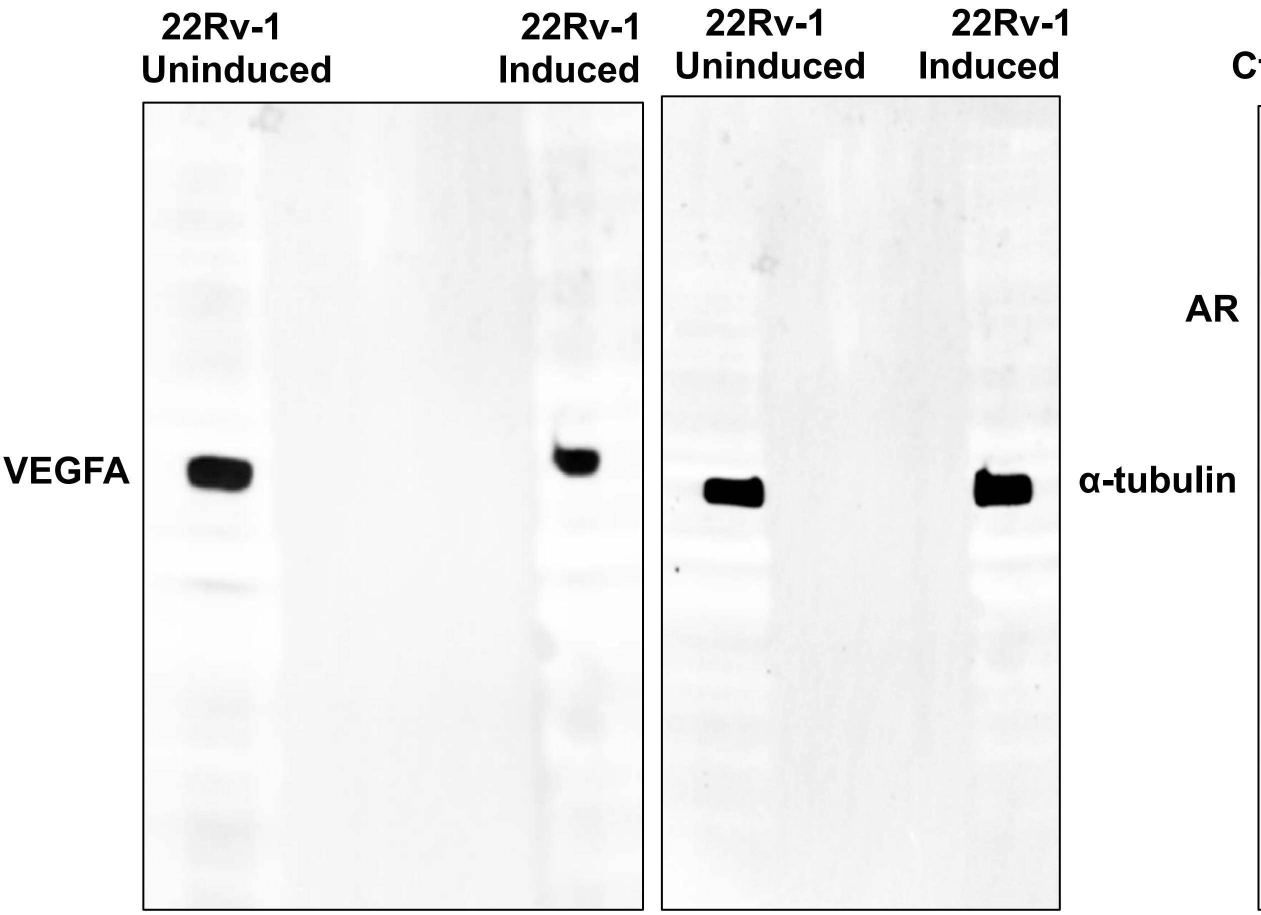

Fig. S5C

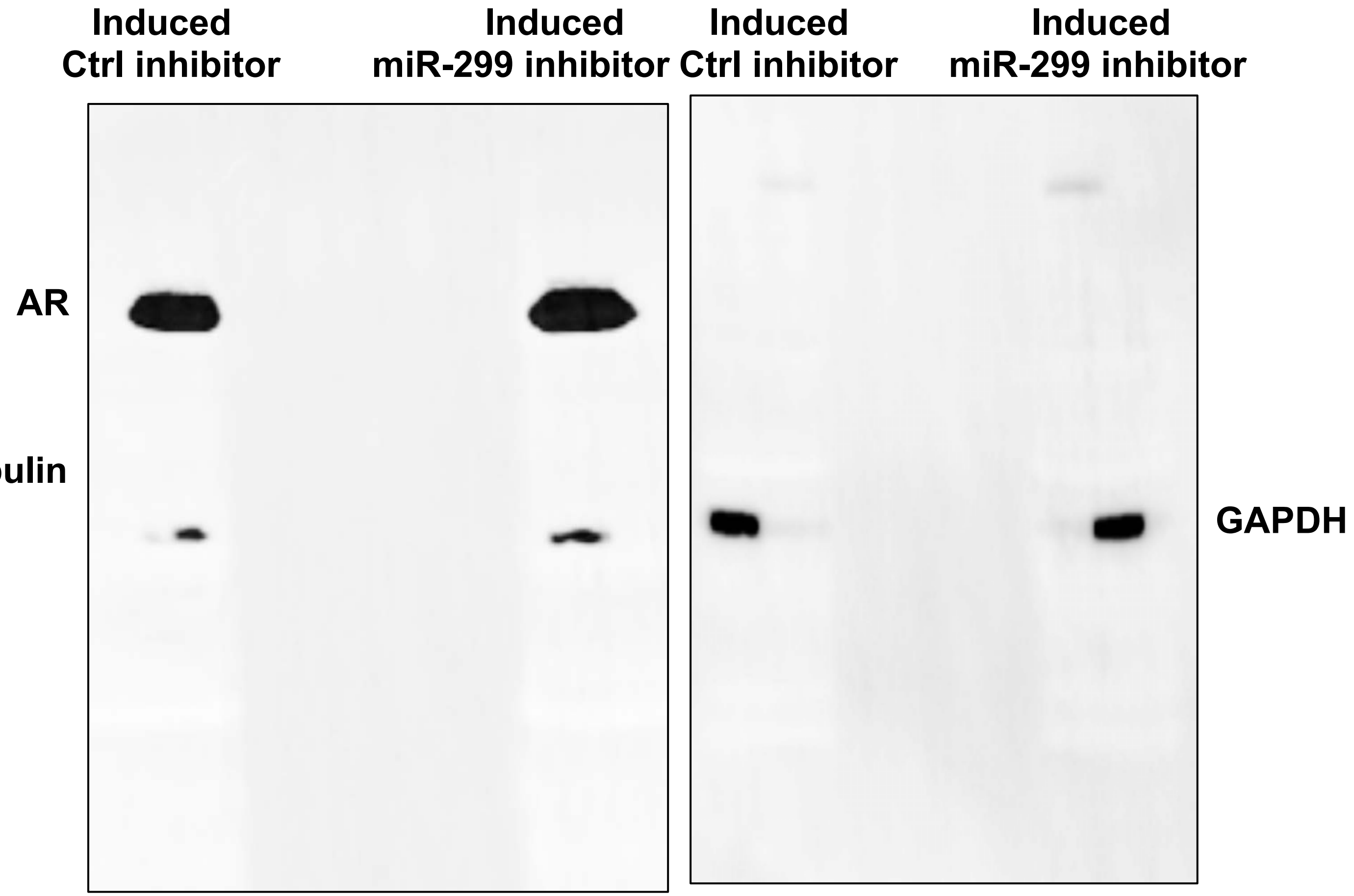

Fig. S5F

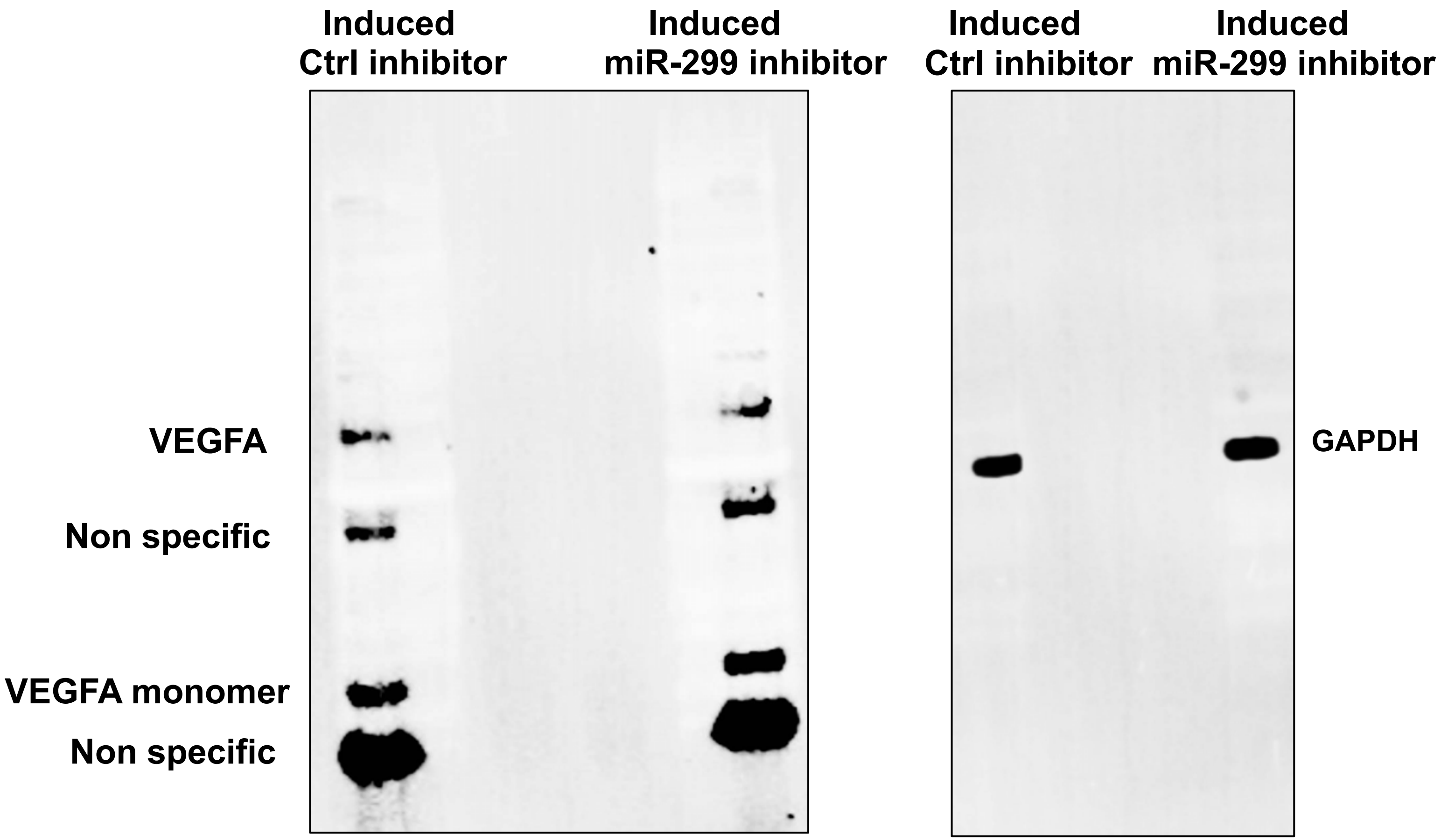
Induced
Ctrl inhibitor
Induced
miR-299 inhibitor
Induced
Ctrl inhibitor
Induced
miR-299 inhibitor
VEGFA
Non specific
VEGFA monomer
Non specific
GAPDH

Fig. S5F

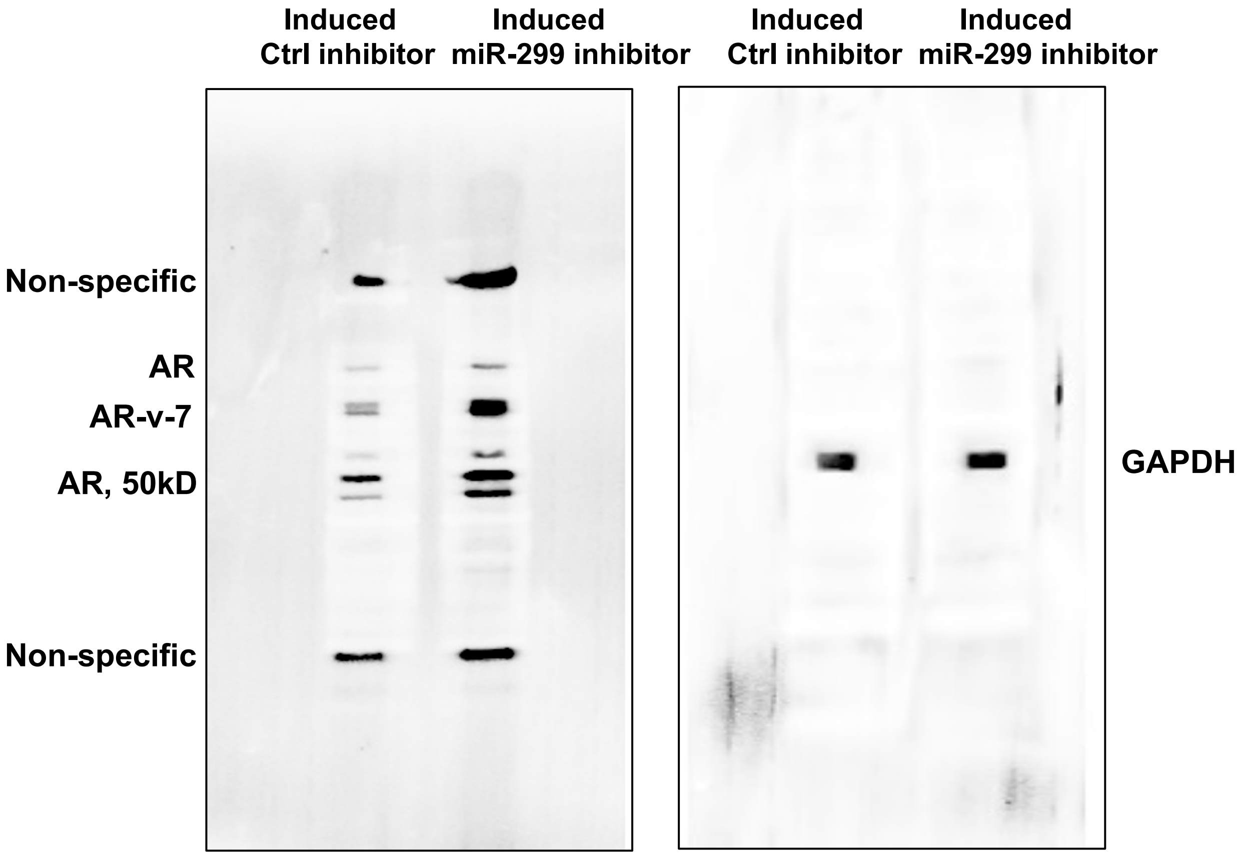
Induced
Ctrl inhibitor
Induced
miR-299 inhibitor
Induced
Ctrl inhibitor
Induced
miR-299 inhibitor
Non-specific
AR
AR-v-7
AR, 50kD
Non-specific
GAPDH

Fig. S5G

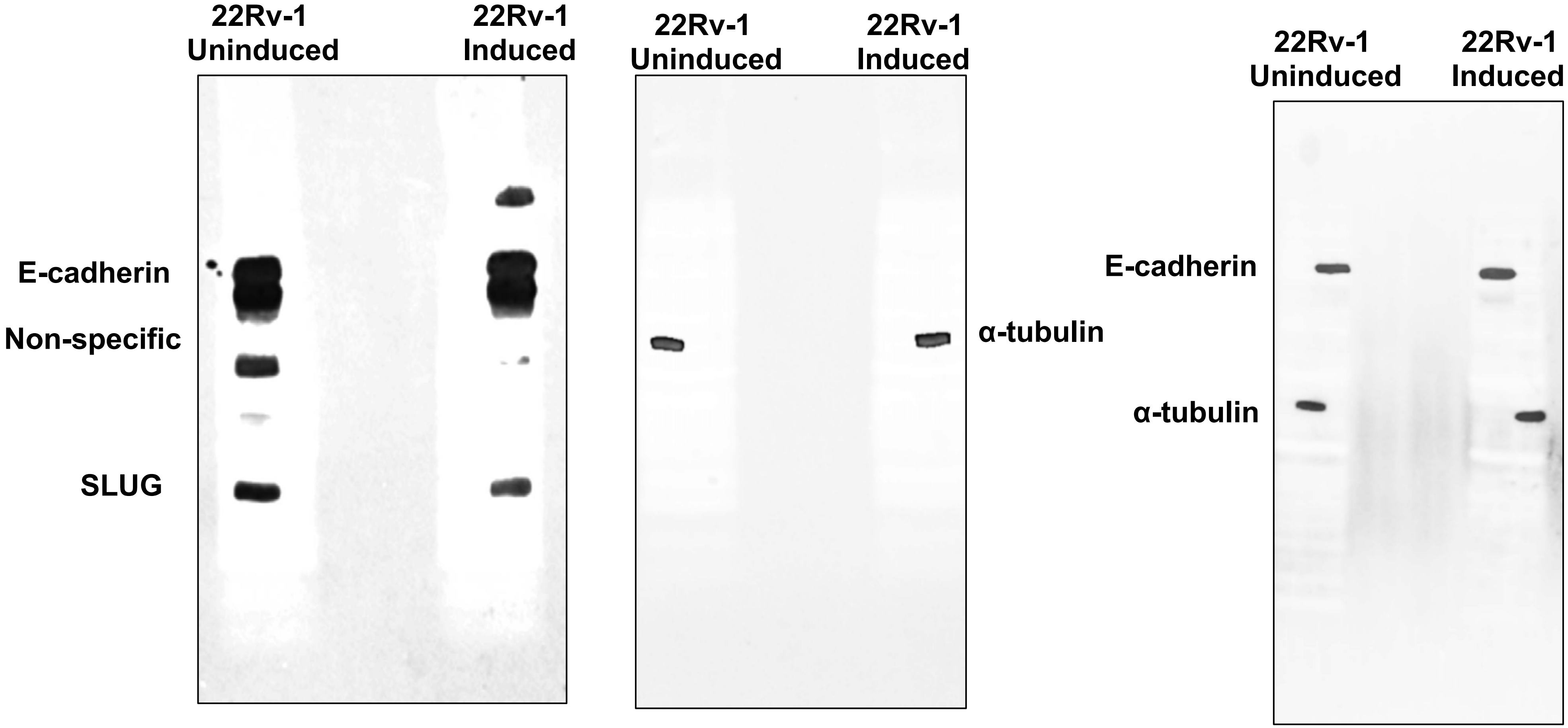

The blot was re-probed for E-cadherin

Fig. S6C
